# Synthesis and Characterization of Ion Pairs between Alkaline Metal Ions and Anionic Anti-Aromatic and Aromatic Hydrocarbons with π-Conjugated Central Seven- and Eight-Membered Rings

**DOI:** 10.3390/molecules25204742

**Published:** 2020-10-15

**Authors:** Jan Bloch, Stefan Kradolfer, Thomas L. Gianetti, Detlev Ostendorf, Subal Dey, Victor Mougel, Hansjörg Grützmacher

**Affiliations:** 1 Department of Chemistry and Applied Biosciences, ETH Zürich, 8093 Zürich, Switzerland; bloch@inorg.chem.ethz.ch (J.B.); krastefa@inorg.chem.ethz.ch (S.K.); detos@gmx.de (D.O.); deys@ethz.ch (S.D.); mougel@inorg.chem.ethz.ch (V.M.); 2 Department of Chemistry & Biochemistry, 1306 E. University Blvd., Tucson, AZ 85719, USA; tgianetti@email.arizona.edu

**Keywords:** aromaticity, ion pairs, alkali metals, tropylidenyl ions, cyclooctatetraene ions, rhodium, electron paramagnetic resonance (EPR) spectroscopy, density functional theory (DFT), electrochemistry

## Abstract

The synthesis, isolation and full characterization of ion pairs between alkaline metal ions (Li^+^, Na^+^, K^+^) and mono-anions and dianions obtained from *5H*-dibenzo[*a*,*d*]cycloheptenyl (C_15_H_11_ = trop) is reported. According to Nuclear Magnetic Resonance (NMR) spectroscopy, single crystal X-ray analysis and Density Functional Theory (DFT) calculations, the trop^‒^ and trop^2−•^ anions show anti-aromatic properties which are dependent on the counter cation M^+^ and solvent molecules serving as co-ligands. For comparison, the disodium and dipotassium salt of the dianion of dibenzo[*a,e*]cyclooctatetraene (C_16_H_12_ = dbcot) were prepared, which show classical aromatic character. A d^8^-Rh(I) complex of trop^−^ was prepared and the structure shows a distortion of the C_15_H_11_ ligand into a conjugated 10π -benzo pentadienide unit—to which the Rh(I) center is coordinated—and an aromatic 6π electron benzo group which is non-coordinated. Electron transfer reactions between neutral and anionic trop and dbcot species show that the anti-aromatic compounds obtained from trop are significantly stronger reductants.

## 1. Introduction

**Scheme 1:**
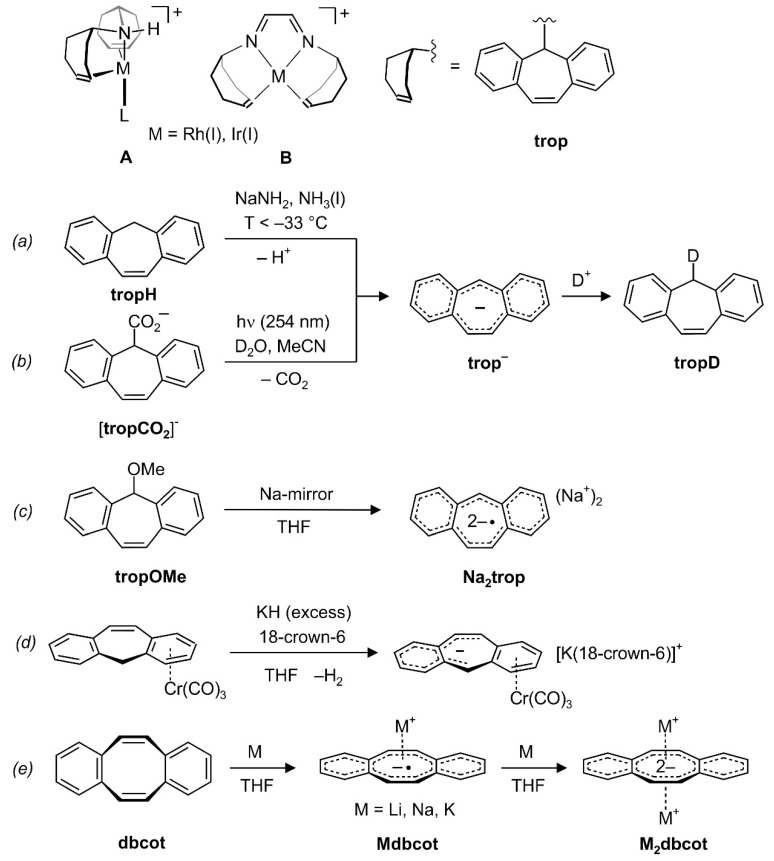
Complexes **A** and **B** with trop amines as ligands. (**a**) and (**b**): In situ generation of the trop anion **trop^‒^** by deprotonation of **tropH** or photochemical CO_2_ extrusion from the carboxylate **tropCO_2_^−^**. (**c**) In situ synthesis of **Na_2_trop**. (**d**) Synthesis of the trop^‒^ complex [K(18-crown-6)][Cr(trop)(CO)_3_]. (**e**) Synthesis of **Mdbcot** and **M_2_dbcot**.

Transition metal complexes with *5H*-dibenzo[*a*,*d*]cyclohepten-5-yl units (trivial name tropylidenyl = trop, see Scheme 1, right) as ligands are well established in the literature [1,2,3,4]. Their special properties give rise to complexes with extraordinary catalytic activities. For example, bis(trop)amine as ligand in d^8^-Rh(I) complex **A** (Scheme 1, top) provokes an unusual butterfly-type structure for tetracoordinated sixteen electron configured transition metal complexes and can act as a cooperating ligand [5]. Both factors contribute to the high activities in the hydrogenation of ketone derivatives [6,7] or the dehydrogenative coupling of alcohols [8,9] under very mild reaction conditions. In another example, complexes of type **B** with the redox and chemically non-innocent cooperative diazadiene ligand trop_2_dad (dad = diazadiene) [10] show an unprecedented high efficiency in dehydrogenation reactions of methanol or formaldehyde [10,11,12]. The olefinic double bond in the trop moiety acts as an electronically flexible ligand [13] and allows to stabilize low-valent metal centers [10].

On the other hand, compounds with main group element metals and trop-type ligands are very scarce [14]. We became especially interested in compounds which contain an alkaline metal ion and an anionic trop moiety because of their potential as building blocks for the synthesis of new trop-type ligands. Furthermore, these species, (M+)_n_[trop]^n−^, may form various forms of ion pairs with fascinating properties, and related species with reduced arenes as anions are interesting to explore on their own [15,16,17].

To the best of our knowledge, salts containing anionic *5H*-dibenzo[*a*,*d*]cycloheptenides, [trop]^n−^ (*n* = 1, 2) have never been isolated. Based on the 4n-Hückel-rule, the trop anion, C_15_H_11_^−^, with its central 8π electron system flanked by two annulated benzo groups (giving a 16π electron system in total), should show anti-aromatic character. Indeed, when the trop anion is generated in situ in liquid ammonia by deprotonation of the neutral hydrocarbon suberene (**tropH**) ((a) in Scheme 1) [18], strongly shielded signals are observed in the ^1^H NMR spectrum, as expected for an anti-aromatic compound [19]. Wan et al. suggested that the photodecarboxylation of the trop carboxylate anion **tropCO_2_^−^** [20,21] and the photochemical hydrogen–deuterium exchange of **tropH** [22,23] both proceed via the trop anion, **trop^‒^**, as intermediate ((b) in Scheme 1). This anion was also generated by deprotonation of suberene with *n*-butyllithium and characterized in situ by Ultraviolet/Visible (UV/Vis) spectroscopy [22,23]. In addition, the reduction of trop methoxide with elemental sodium was reported to give the trop dianion radical, C_15_H_11_^2−•^ ((c) in Scheme 1), which was characterized in situ by Electron Paramagnetic Resonance (EPR) spectroscopy [24].

The only compound we are aware of in which **trop^−^** acts as a ligand to a transition metal was reported by Venzo et al., which showed that in complex **C**, the paratropic character of the trop anion significantly decreases upon coordination to a tricarbonylchromium group [25]. Based on ^1^H and ^13^C NMR spectroscopy, the authors suggested that the anti-aromatic 16π-electron structure is split into a 6π-electron system located on one of the benzo groups which coordinates to the Cr(CO)_3_ fragment and an uncoordinated 10π-electron system, as shown in (d) in Scheme 1 [25,26].

Cyclooctatetraene, C_8_H_8_ (cot), is a textbook example in which the anti-aromatic character of a hypothetical planar molecule with an 8π-electron system leads to a severe structural distortion into a tub conformation. The same is true for its tub-shaped benzoannulated derivative dibenzo[*a*,*e*]cyclooctatetraene, **dbcot**, with alternating single and double bonds ($\bar{\Delta C=C}$ = 0.1170(10) Å) [27]. Reduction of **dbcot** with alkaline metals Li, Na and K ((e) in Scheme 1), proceeds stepwise to first give **Mdbcot** containing the dbcot^−•^ anion radical and then to M_2_dbcot with the dbcot dianion (*vide infra*) [28,29]. The EPR spectra of **Mdbcot** suggest that there are contacts between the cations M^+^ (M = Li, Na, K) and dbcot^−•^ [28,30]. Only the structure of the ion triple [Li(tmeda)]_2_[dbcot] (tmeda = tetramethylethylenediamine) was determined. In this ion triple, the dianion adopts a planar structure with nearly equal C-C bond lengths in the central ring (the average bond length variation amounts to $\bar{\Delta C=C}$ = 0.024(2) Å). The Li(tmeda)^+^ units are η^8^-bound above and below the center of the dianion [31]. The ^1^H NMR spectrum of the dianion shows that the proton resonances are significantly de-shielded. For example, the olefinic resonances in the central C8 ring are shifted from 6.72 ppm in dbcot [32] to 7.08 ppm in **Li_2_dbcot** [28]. In combination with the structural data, the dbcot^2−^ dianion is therefore considered to be aromatic [19].

Herein, we report a new simple and clean synthesis of Mtrop and M_2_trop which allows the isolation of various close and separated ion pairs which contain the trop mono- and di-anion. For comparison, the disodium and dipotassium salts of dbcot^2−^ were also prepared and structurally characterized. Their anti-aromatic or aromatic character is assessed experimentally based on structural and magnetic criteria. DFT-calculations on simplified model compounds, specifically calculation of the Nuclear Independent Chemical Shifts (NICS), were performed in order to bolster the conclusions derived from the experimental data.

## 2. Results

**Scheme 2:**
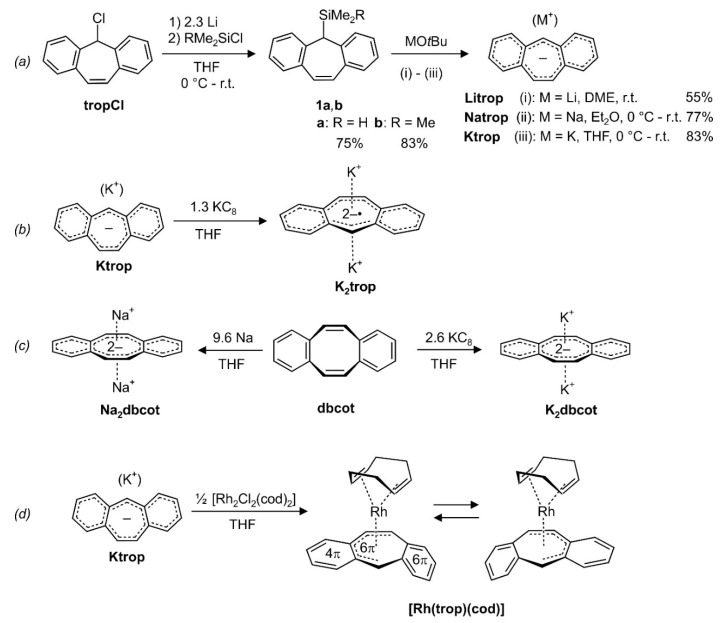
(**a**) Synthesis of the silylated tropylidenes 1a, b and the trop^‒^ containing ion pairs Mtrop (M = Li, Na, K). (**b**) Synthesis of trop^2−•^ dianion radical containing ion triple K_2_trop by reduction of Ktrop. (**c**) Synthesis of the dbcot^2‒^ dianion containing ion triples M_2_dbcot (M = Na, K) by reduction of dbcot. (**d**) Synthesis of the organometallic d^8^-Rh(I) complex [Rh(trop)(cod)]. r.t. = room temperature; DME: 1,2-dimethoxyethane; Et_2_O: diethyl ether; THF: tetrahydrofuran.

In order to obtain the trop anion trop^–^ in pure form, the silylated trop derivatives **1a** (R = H) [3] or **1b** (R = Me) (**2**) were prepared by reacting tropCl [33] with an excess of Li and quenching the trop^‒^ as appearing intermediate with HMe_2_SiCl or Me_3_SiCl ((a) in Scheme 2). Both 1a and 1b can be easily purified and obtained in pure form as crystalline solids [3]. The trop silanes were subsequently treated with various alkali metal *tert*-butoxides to give the ion pairs **Litrop**, **Natrop** and **Ktrop** respectively, with trop^‒^ as the anion, as pure crystalline solids in moderate to good yields ((a) in Scheme 2).

Compound **Ktrop** can be further reduced with potassium graphite to give the ion triple **K_2_trop**, which, apart from two K^+^ ions, contains the trop dianion radical trop^2−^**^•^** ((b) in Scheme 2). **K_2_trop** was isolated as a dark green, micro-crystalline solid. We failed to prepare and isolate the dilithium and the disodium analogue so far. The synthesis of the disodium and the dipotassium derivatives of the dbcot^2−^ dianion, **Na_2_dbcot** or **K_2_dbcot**, is achieved in a straightforward manner by simply exposing dibenzo[*a*,*e*]cyclooctatetraene (dbcot) to elementary sodium or potassium graphite in anhydrous tetrahydrofuran ((c) in Scheme 2) as was likewise reported for the preparation of **Li_2_dbcot** [28,31].

The trop anion can be used as a ligand in transition metal complexes, and reaction with half an equivalent of [Rh_2_Cl_2_(cod)_2_] gives **[Rh**(**trop**)(**cod**)**]** as a dark red crystalline compound in moderate isolated yield (63%) ((d) in Scheme 2). In this complex, the d^8^-valence electron configured Rh(I) center is exclusively coordinated to hydrocarbon ligands and represents to the best of our knowledge the first fully characterized mononuclear Rh(I) heptatrienide species. Only very few related compounds such as bimetallic Rh complexes [34,35], a Rh(I) azulene complex [36] and a Rh(III) cycloheptatrienide complex have been reported [37]. In contrast, the reaction with the dianion radical containing salt **K_2_trop** did not yield any identifiable compound, likely due to the strongly reducing properties of the trop^2−•^ (*vide infra*).

### 2.1. Characterization by NMR, EPR and X-ray Diffraction Methods

All diamagnetic compounds were fully characterized by NMR spectroscopy. The paramagnetic compound **K_2_trop** was analyzed by EPR spectroscopy. Single crystals of **Litrop**, **Natrop**, **Ktrop**, **K_2_trop**, **Na_2_dbcot**, **K_2_dbcot** and **[Rh**(**trop**)(**cod**)**]** were grown and investigated with X-ray diffraction methods in order to determine the structures experimentally. In addition, the structures of compounds **Mtrop** (M = Li − K) and **[Rh**(**trop**)(**cod**)**]** and their degree of anti-aromatic or aromatic character was evaluated by calculating the Nuclear Independent Chemical Shifts (NICSs) (DFT, PBE [38,39] and 6-311+G (df, pd) [39] in a continuum solvation model (THF) [40,41]).

All compounds containing the trop^‒^ anion show strongly shielded signals for all protons in the ^1^H NMR spectra. In the dimer of the trop radical [23], namely C_15_H_11_ − C_15_H_11_ = trop_2_ [42] (entry 1, Table 1), which has an electronic structure reminiscent of the conjugated hydrocarbon *cis*-stilbene, the olefinic protons ^1^H_ol_ and the benzylic proton ^1^H_bz_ attached to the central seven-membered ring are observed in the normal range at δ = 7.06 ppm and δ = 4.73 ppm. In the ion pairs, **Mtrop**, these resonances are significantly shifted to lower frequencies to δ (^1^H_ol_) < 2.6 ppm and δ (^1^H_bz_) < 0.56 ppm (M = Li, Na, K, entries 2–4, Table 1). Also, the protons of the annulated benzo groups are strongly shifted to lower frequencies by about 3 ppm.

**Figure 1:**
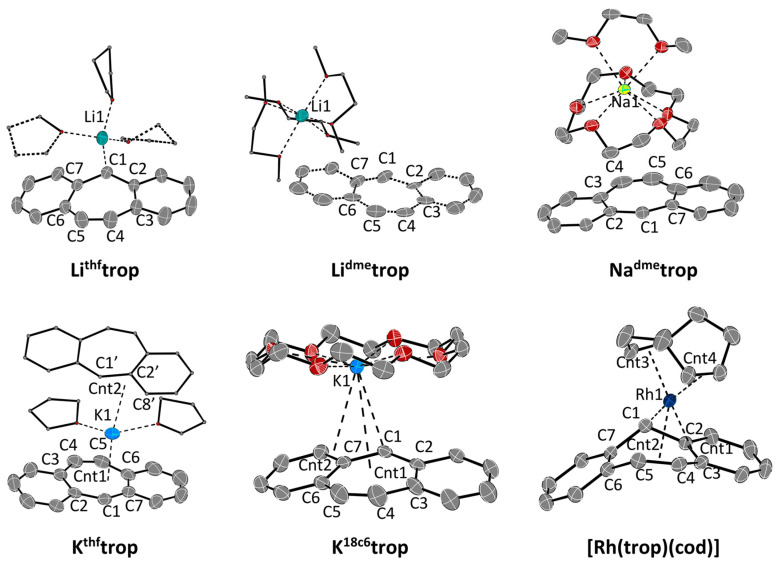
ORTEP plots **Li^thf^trop**, **Li^dme^trop**, **Na^dme^trop**, **K^thf^trop**, **K^18c6^trop** and **[Rh**(**trop**)(**cod**)**]** at 50% ellipsoid probability (hydrogen atoms are omitted for clarity). **Li^dme^trop**: only one of the two distorted trop moieties is shown. **[Rh**(**trop**)(**cod**)**]**: A co-crystallized toluene-molecule is omitted for clarity. Selected Rh-C bond distances [Å]: Rh1–C1 2.0912(16), Rh1–C2 2.3427(16), Rh1–C3 2.3995(16), Rh1–C4 2.2396(17), Rh1–C5 2.4044(18). Further selected structural parameters of all compounds are given in Table 2 and Table 3.

The experimental structures of the contact ions pairs **Li^thf^trop**, **Li^dme^trop, Na^dme^trop**, **K^thf^trop** and **K^18c6^trop** were determined with X-ray diffraction methods using a suitable single crystal of every compound and are depicted in Figure 1. Selected bond distances and angles are listed in Table 2 and Table 3. The most remarkable feature is the bending of the central seven-membered ring in the trop^‒^ moiety expressed by the fold angles *Θ*_1_ and *Θ*_2_ (Table 3). These angles vary strongly with the nature of the counter cation but also with the nature of the solvent molecules acting as co-ligands to M^+^. In **Li^thf^trop**, the [Li(thf)_3_]^+^ cation binds predominantly to C1 [Li-C1 2.279(3) Å] which provokes a rather strong bending (*Θ*_1_ = 24.2°; *Θ*_2_ = 14.1°), about half of the one observed in the hydrocarbon trop_2_ (*Θ*_1_ = 49.4°; *Θ*_2_ = 22.5°). Encapsulation of Li^+^ by three dimethoxyethane (dme) solvent molecules leads to [Li (dme)_3_]^+^[trop^−^] (**Li^dme^trop**), in which no close contact between cation and anion occurs (shortest Li–C contact = 6.79 Å) and the trop^‒^ anion adopts an almost flat structure (*Θ*_1_ = 6°; *Θ*_2_ = 3°). Likewise, in the structure of **Natrop**, which contains [Na(dme)_3_]^+^, the contact between cation and anion is long (the distance between Na^+^ and the centroid (cnt) of the central C_7_ ring is 5.25 Å) and the trop^‒^ anion is flat (*Θ*_1_ = 6.5°; *Θ*_2_ = 4.7°).

The most planar structure for a trop^‒^ anion is observed in **K^thf^trop** (*Θ*_1_ = 1.7°; *Θ*_2_ = 2.4°), which is best described as a close ion pair with a rather short distance between K1 and the centroid cnt1 of the C_7_ ring of 2.82 Å. The K^+^ is coordinated to the C_7_ ring in a slightly asymmetric manner as indicated by the unequal K-C1 (3.098 Å) and K-C4/C5 distances (3.407 Å; 3.346 Å). Furthermore, the potassium ion has an additional contact at about 2.97 Å to another trop^‒^ unit, such that K^+…^trop^−…^K^+….^trop^−^ chains are formed in the solid state. Remarkably, in **K^18c6^trop**, the trop^−^ anion shows larger fold angles (*Θ*_1_ = 12.4°; *Θ*_2_ = 12.3°). In this ion pair, K^+^ is rather asymmetrically bound and the distance to the centroid of to the central C_7_ ring is large (3.812 Å). Instead, K^+^ binds closer to the allylic fragment C1, C7, C15 (K1-cnt2 3.303 Å) with a rather short contact to the benzylic carbon center C1 (3.02 Å) and longer ones to C7 (3.263 Å) and C15 (3.490 Å). It is highly speculative to which extent these features of the solid-state structures are retained in solution. But, the coupling constants ^1^*J*_HC_ of the benzylic carbon nucleus, ^13^C_bz_, to the benzylic proton, ^1^H_bz_, can be empirically correlated to the s-orbital character in the C_bz_–H bond. In a bent trop anion, the C_bz_–H bond will be closer to a sp^3^ configuration (25% s, smaller ^1^*J*_HC_) that in a flat structure, where the C_bz_–H is closer to a sp^2^ configuration (33% s, larger ^1^*J*_HC_). Indeed, ^1^*J*_HC_ obtained in deuterated tetrahydrofuran, [D_8_]THF, as solvent (Table 1) is larger for the potassium salt than in the bent lithium salts, indicating that the solid-state structures are retained to a certain degree in solution.

At this point, it is interesting to compare these main group metal complexes of trop^−^ with a “classical” organometallic complex such as **[Rh**(**trop**)(**cod**)**]**. In contrast to **Mtrop**, which shows strongly shielded ^1^H NMR resonances for all protons, the signals for the ^1^H nuclei at the benzo groups in **[Rh**(**trop**)(**cod**)**]** are in the same region (δ ^1^H: 6.82–7.01 ppm) as in the reference compound **trop_2_** (δ ^1^H: 6.58–7.25 ppm). Also, the chemical shifts of the benzo ^13^C nuclei are in the normal range for arenes in between δ = 127 and 137 ppm. Only the olefinic and benzylic ^1^H and ^13^C NMR signals (δ^1^H_ol_, δ^1^H_bz_, δ^13^C_ol_, δ^13^C_bz_) are significantly shifted to lower frequencies, indicating an interaction with the metal center, c.f. δ (^13^C_ol_) = 97.6 ppm, δ (^13^C_bz_) = 56.5 ppm in **[Rh**(**trop**)(**cod**)**]** vs. δ (^13^C_ol_) ~139 ppm and δ (^13^C_bz_) = 81.6–89.4 ppm in **Mtrop**, see Table 1). In solution, **[Rh**(**trop**)(**cod**)**]** is seemingly *C_2v_*-symmetric which is not in agreement with the structure in solid state but can be explained by a dynamic phenomenon (*vide infra*). Complex **[Rh**(**trop**)(**cod**)**]** crystallizes as racemic mixture in the space group P2_1_/c. The structure of one of the enantiomers is depicted in Figure 1 and is very different from the **Mtrop** compounds. The Rh(I)(cod) fragment binds to C1, C2, C3, C4 and C5 at distances between 2.09 to 2.40 Å (see legend to Figure 1) of the central seven-membered ring in a way which is also observed in other metal [43,44] and specifically, Rh(I) pentadienide complexes [43,44]. This pentadienide-type interaction donates 6π electrons to the Rh(I)(cod) fragment which thereby reaches an 18-electron configuration. Metal-to-ligand electron back donation leads to a slight elongation of all involved C–C bonds by about 0.03 Å with respect to the distances in the trop anion in the **Mtrop** contact pairs (M = Li, Na, K). The most notable structural feature is the strongly bent conformation of the trop^‒^ unit: the intersection angle between the non-coordinated benzo group and the benzopentadienide unit is *θ*_3_ = 49.9(1)° (cf. Figure 1). The *η^5^* coordination mode and non-symmetric structure observed in the solid state is in contrast with the apparent *C_2v_* symmetry of the complex in solution. We therefore assume that in solution, **[Rh**(**trop**)(**cod**)**]** underlies a dynamic phenomenon by which the rhodium center rapidly exchanges between the two possible *η^5^* binding sites of the central seven-membered ring, as shown in Scheme 2d. This process must have a very low activation barrier because even at low temperatures, this process is not frozen out on the NMR time scale. This coordination mode is in stark contrast to the one of the only other dibenzo[*a*,*d*]cycloheptenide metal complex **C** (Scheme 1d) [25,26]. Here, NMR data indicate that the Cr(0)(CO)_3_ fragment binds in a *η^6^* fashion to one of the annulated benzo groups of the trop moiety which leads to C_1_ symmetric structure. The ^13^C chemical shifts of this coordinated benzo group are characteristically shifted to lower frequencies [δ(^13^C) = 70–100 ppm]. That is, in this case, the anti-aromatic π-electron system of trop^‒^ is localized in a different way from the one in **[Rh**(**trop**)(**cod**)**]**, namely into a 6π-electron system at a terminal arene bound to the metal and an uncoordinated 10π-electron system.

**Figure 2:**
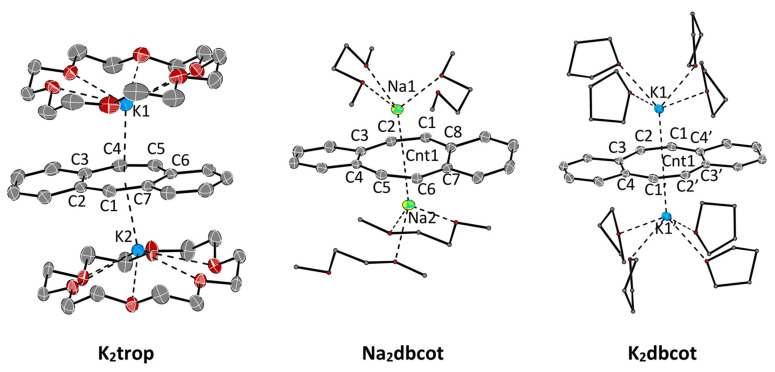
ORTEP plots of dianions at 50% ellipsoid probability (hydrogen atoms are omitted for clarity). **K_2_trop**: A co-crystallized DME-molecule is omitted for clarity. **Na_2_dbot**, **K_2_dbot**: The second chemically identical but crystallographically unique compound in the asymmetric unit is omitted for clarity. Selected bond lengths (Å) and angles (°) are shown in Table 3 and Table 4.

The structures of the ion triples **K_2_trop**, **Na_2_dbcot** and **K_2_dbcot** with the trop^2‒•^ dianion radical or the dbcot^2‒^ dianion respectively, are shown in Figure 2. Selected bond lengths and angles are listed in Table 3 and Table 4.

As expected, the reduction of **Ktrop** to the radical dianion **K_2_trop** leads to an overall elongation of all bonds within the central seven-membered ring (see increased average bond length $\bar{C=C}$ in Table 3). The average bond length difference ($\bar{\Delta C=C}$ in Table 3) becomes smaller and the C3–C4, C4–C5 and C5–C6 bond lengths’ alternation is slightly diminished. The plots and structural data for **Na_2_dbcot** and **K_2_dbcot** are given in Figure 2 and Table 4 and are very similar to the ones reported for **Li_2_dbcot** (Scheme 1 and Table 3) [31]. The dbcot^2‒^ dianions in **K_2_dbcot** and **Li_2_dbcot** adopt a rather flat conformation while the one in **Na_2_dbcot** shows a slightly twisted conformation likely because of crystal packing forces (see dihedral angles $\varphi_{1}$ and $\varphi_{2}$ in Table 3). All C–C bond lengths are longer than 1.4 Å and there is very little bond length variation (see $\bar{\Delta C=C}$ in Table 3).

**Figure 3:**
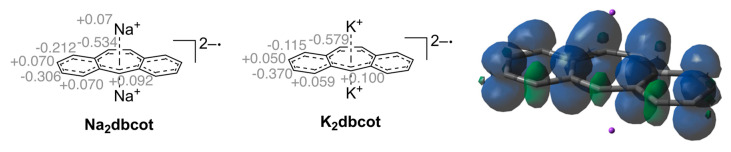

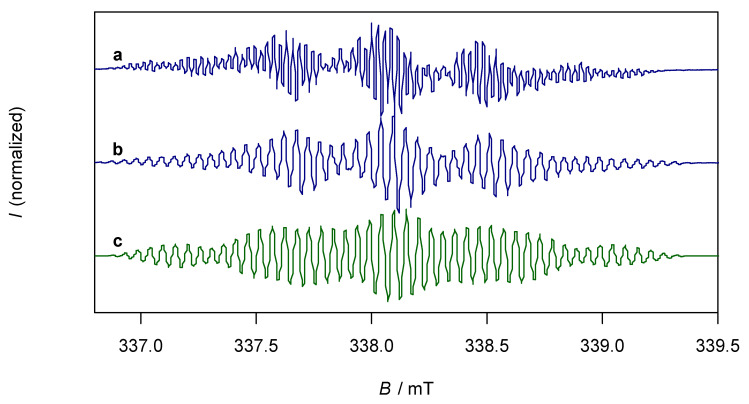
Top left: Reported hyperfine splittings (mT) of **Na_2_trop**. Top middle: Calculated hyperfine coupling constants (HFCs) (mT) from the simulated spectrum of **K_2_trop**. Top right: Spin density plot of **K_2_trop** (isovalue = 0.0009, positive spin densities in blue and negative spin densities in green). DFT calculation: PBE [38] 6-311+G(df, pd) [39] in a continuum solvation model (THF). Bottom (**a**): EPR spectrum of **K_2_trop** (0.5 mM) in THF, g = 2.0064. (**b**): EPR spectrum of **K_2_trop** (0.5 mM) and 18-crown-6 (2 equivalents) in THF, g = 2.0067. (**c**): Simulated spectrum of **b**.

The paramagnetic compound **K_2_trop** was further characterized by EPR spectroscopy. In tetrahydrofuran (THF) as solvent, the EPR spectrum of **K_2_trop** shows a rather complex hyperfine coupling pattern (Figure 3, line a) which can be simplified by the addition of two equivalents of 18-crown-6. This indicates that complexation of K^+^ by 18-crown-6 significantly elongates the distances between the cation and dianion radical, which leads to much smaller and hence non-detectable ^39^K-hyperfine splittings. Indirect proof comes from a comparison of the structures **K_2_trop**, where one 18-crown-6 binds to every K^+^ ion, and **K_2_dbcot**, where each K^+^ ion is coordinated by four THF molecules. In the former the K^+^-Cnt distance is 2.79 Å while in the latter a shorter distance of 2.47 Å is observed (Cnt = centroid of the central ring). The less complex spectrum (Figure 3, line b) was used to simulate the hyperfine pattern (Figure 3, line c). The experimentally determined and calculated hyperfine coupling constants (HFCs) indicate delocalization of the unpaired electron over the whole dianion (see spin density plot in Figure 3). These data and the corresponding g-factor of 2.0064 are in fairly good agreement with the ones of the reported sodium analogue **Na_2_trop** [24] more than 50 years ago (g = 2.0027 and HFCs shown in Figure 3).

The ^1^H NMR spectra of the contact ion pairs **M_2_dbcot** (M = Na, K) show, in comparison to neutral dbcot, which is best described as a cyclic conjugated polyolefin, a diatopic ring current which leads to significantly de-shielded olefinic resonances, ^1^H_ol_ > 7 ppm (Table 1).

### 2.2. Evaluation of Anti-Aromaticity and Aromaticity by Calculation of NICS Values

Table 5 lists the calculated nuclear independent chemical shifts in the center of the rings, NICS(0), and 1 Å above and below the central plane of these, NICS(1) (DFT, PBE [38] 6-311+G(df, pd) [39]). The data for both six-membered rings A and C and the central seven-membered ring B is given. For comparison, also the data for the tropylium cation **trop^+^** are listed. As expected for an aromatic molecule with a 14 π-electron configuration, the NICSs data are strongly negative for all rings in **trop^+^**. On the other end of the scale, cyclobutadiene (CBD) is listed for comparison which is considered to be an archetypical anti-aromatic molecule with a 4-electron system and shows positive NICS values ((NICS(0) = 27.49, NICS(1) = 18.03). The values are largely exceeded by the NICS values of the central ring B in the flat and counter cation-free trop anion **trop^‒^** which amount to 45.00 and 34.54. Even the flanking rings A and C in **trop^‒^** reach positive values in the range of CBD. For simplicity, the NICS data were calculated for simplified model compounds of the contact ion pairs **Mtrop^m^** (M = Li, Na, K; m indicates neglecting coordinated solvent molecules at M^+^) and **K_2_trop^m^**, where the solvent molecules bound to M^+^ were not considered. Nevertheless, the calculated π–π* transitions of the calculated UV/Vis spectra correspond well to the recorded UV/Vis spectra (Supplementary Figures S1–S4)—going from Li to K, the observed bathochromic shift is for example well reproduced by the model compounds. In addition, the overall structural agreement between these model compounds and the experimentally determined structures given in Table 3 is very good (e.g., the calculated distances C5-M are listed in Supplementary Table S1) and specifically, the decrease of the bent angles in the order **Litrop^m^** > **Natrop^m^** > **Ktrop^m^** is well reproduced. The NICS values of the contact ion pairs are very sensitive to the bending of the central seven-membered ring. The stronger the ring B is bent, as in **Litrop^m^** (*θ*_1_ = 26.2°, *θ*_2_ = 13.5°), the smaller the NICS values are and the less pronounced the anti-aromatic character. On the contrary, when the bending becomes small such as in **Ktrop^m^**, the NICS values reach the ones of flat **trop^‒^**. While it may be debated whether CBD is an anti-aromatic compound because of its rectangular distortion [46], **Ktrop^m^** can be truly considered as an anti-aromatic species which in the form of **K^thf^trop** can be isolated as a substance. This claim is further bolstered by the fact that the averaged experimental C=C bond distances listed in Table 3 do not differ much between **trop^+^** and **K^thf^trop** and the C=C bond length variation ΔC=C is modest (0.028 Å in **trop^+^**, vs. 0.049 Å in **K^thf^trop**)**,** indicating only small structural distortions. This is in contrast to CBD, where this variation is significant (C-C 1.576 Å, C=C 1.332 Å, $\bar{\Delta C=C}$ = 0.244 Å) [46].

As already stated above, the trop^‒^ anion as a ligand in a transition metal complex behaves very differently. The NICS data of the annulated benzo rings A and C in **[Rh**(**trop**)(**cod**)**]** are negative and indicate modest magnetic aromatic character.

The NICS data for the ion triple containing paramagnetic dianion radical trop^2‒•^ are much smaller than for the mono-anions and indicate diminished anti-aromatic character in accord with conclusions derived from a comparison of the experimental structural data (the C=C bond lengths variation is slightly diminished in the less anti-aromatic dianion radical, see Table 3).

For completeness, the NICS(0) and NICS(1) data for the free dianion **dbcot^2‒^** and the model compounds **M_2_dbcot^m^** (M = Li, Na, K) are listed in Table 6. As expected, all rings A, B, and C show negative NICS values indicating aromatic character. Note that the structural parameters and the UV/Vis spectra of the ion triples are accurately reproduced by DFT-calculations (Supplementary Figures S5–S7).

### 2.3. Electrochemistry

**Figure 4:**
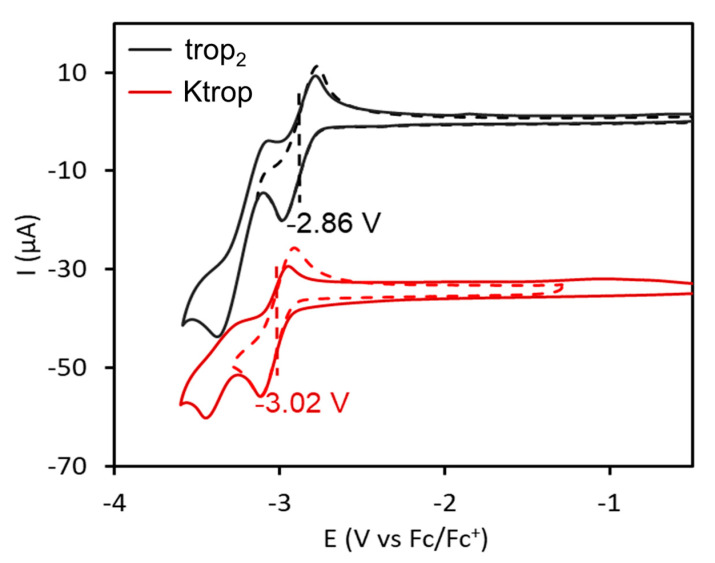
Cyclic voltammogram of **trop_2_** (1 mM) in dry tetrahydrofuran at ambient temperature with scan rates ranging from 50 to 400 mV/s and referenced vs. Standard Calomel Electrode (SCE) [48]. [(*n*Bu)_4_N][PF_6_] (100 mM) was used as a supporting electrolyte.

The electrochemical properties of **trop_2_**, **Ktrop** and **dbcot** were investigated to provide some understanding of the reactivity, especially with respect to mutual electron exchange processes. As previously reported, the cyclic voltammogram of **dbcot** shows two close lying reduction events at E° = −2.24 V and E° = −2.33 V vs. Fc/Fc^+^, resulting from two consecutive one-electron transfer steps to give a planar dbcot^2−^ dianion (Fc = ferrocene, Fc^+^ = ferrocenium) [28,29]. The dbcot^‒•^ anion radical was detected by EPR at low temperatures but cannot be isolated. The formation of the trop^2−•^ dianion radical using **Ktrop** as starting material was investigated electrochemically and the cyclic voltammogram in a 0.1 M tetrabutylammonium hexafluorophosphate (TBAPF_6_) solution in THF is presented in Figure 4. It shows two well-defined waves at −3.04 and −3.37 V vs. Fc/Fc^+^. The redox process at −3.04 V is fully reversible, as indicated by the linear dependence of the anodic and cathodic peak heights as function of ν^1/2^ (Supplementary Figure S8). This reduction was determined to be a one-electron reduction and attributed to the reduction of trop^‒^ to the trop^2−•^ dianion radical. The second process at −3.37 V is irreversible and no anodic wave is recorded upon reversal of the potential scan,, even at high scan rates (this could correspond to the formation of the trianion which was reported for [C_7_H_7_]^3‒^ [47]). The dependence of the cathodic peak current height of this process with ν^1/2^ is below the theoretical curve for a one electron Nernstian reaction (Supplementary Figure S9). In addition, the cathodic peak current decreases upon multiple cycling, while a new oxidation wave at –0.72 V appears, increasing upon multiple cycling (Supplementary Figure S10). This behavior is expected for a redox process coupled to a chemical reaction (EC mechanism) resulting in the deposition of the electrochemically generated species on the electrode. Indeed, a blue film formed after electrolysis on the electrode surface. The identity of that deposit could so far not be determined. The neutral hydrocarbon **trop_2_** shows a comparable behavior. Two consecutive redox waves at –2.86 and –3.29 V vs. Fc/Fc^+^ (Figure 4) are observed in its cyclic voltammogram. The first occurs at a significantly more anodic potential than for the potassium salt **Ktrop** (ΔE = 180 mV). The redox process at −2.86 V is fully reversible whereas the second redox wave at −3.29 V is irreversible. Chronoamperometry and linear sweep voltammetry data indicate that the first reduction process at −2.86 V is a 1 e^–^ reduction (see Supplementary for details, Figures S11–S12 and Table S2). In contrast to the reduction of trop^–^, the variation of the cathodic peak current height of this process with ν^1/2^ follows the theoretical curve for a two-electron Nernstian reaction at low scan rates and a one-electron Nernstian reaction at high scan rates, suggesting an electron transfer-chemical reaction-electron transfer (ECE) mechanism (Supplementary Figure S9). The reversibility of the first reduction process at −2.86 V suggests therefore that the singly reduced radical trop_2_^–•^ has a certain lifetime under electrochemical conditions. The ECE mechanism observed for the second reduction to the dianion trop_2_^2‒^ suggests the cleavage of the C–C bond of trop_2_^2‒^, forming two equivalents of the trop^–^ mono-anion, which are further reduced at these potentials to give the dianion radical trop^2−•^. Hence, the electrochemical behavior of trop_2_ can be summarized with the steps (1)–(4) indicated below:

trop_2_ + 1e^–^ → trop_2_^–•^ (1)

trop_2_^–•^ + 1e^–^ → trop_2_^2−^ (2)

trop_2_^2−^ → 2 trop^–^ (3)

2 trop^–^ + 2 e^–^ → 2 trop^2−•^ (4)

Interestingly, cyclic voltammetry studies of **trop_2_** in the presence of 10 equivalents of KPF_6_ showed anodically shifted redox waves, indicating that potassium ions contribute in stabilizing the trop anions and in facilitating the C–C bond cleavage of trop_2_ (Supplementary Figures S13–S14).

**Scheme 3:**
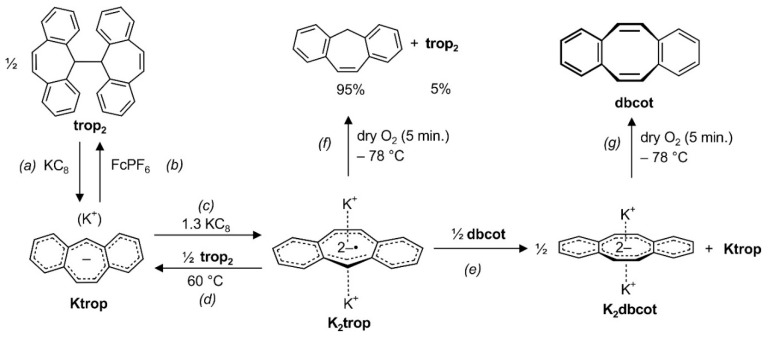
Interconversions reactions between and within the trop and the dbcot scaffold. FePF_6_: ferrocenium hexafluorophosphate.

The electrochemical behavior of **trop_2_** and **Ktrop** is reflected in the chemical reactivity of these species. First, the neutral hydrocarbon **trop_2_** is cleanly converted with KC_8_ under cleavage of the central C–C bond to give **Ktrop** (step (a) in Scheme 3). This reduction is reversible and addition of the ferrocenium salt FcPF_6_ oxidizes **Ktrop** to reform **trop_2_** (step (b) in Scheme 3). As already shown in Scheme 2, **Ktrop** is further reduced to **K_2_trop** with potassium graphite (see (c) in Scheme 3). The anti-aromatic radical dianion salt **K_2_trop** is a strong reductant (E° = –3.04 V). Addition of half an equivalent **trop_2_** oxidizes **K_2_trop** to give the mono-anion **Ktrop**, although heating to 60 °C is required ((d) in Scheme 3). Upon addition of half an equivalent of **dbcot** to **K_2_trop**, the former is fully reduced to the aromatic dianion **K_2_dbcot** ((e) in Scheme 3).

When one equivalent of **dbcot** is used in the oxidation of the dianion radical **trop^2‒•^**, a mixture of **K_2_dbcot** (0.65 equivalents), aside 0.64 equiv. **Ktrop** and 0.16 equiv. of **trop_2_** is obtained according to ^1^H NMR spectroscopy. A featureless EPR signal (g = 2.00485) suggests that also K^+^[dbcot^‒•^] may be present [28,30,49]. Considering the stoichiometry of the reaction, we suspect that K^+^[dbcot^‒•^] (≈0.06 equiv.) is in a rapid equilibrium with the remaining neutral **dbcot** molecules (≈0.3 equiv.) which we could not detect by ^1^H NMR spectroscopy. We also reacted two equivalents of the anti-aromatic mono-anionic ion pair **Ktrop** with **dbcot**. Also, in this reaction, a product mixture consisting of **K_2_dbcot** (0.5 equiv.), unreacted **Ktrop** (0.9 equiv.) and **trop_2_** (0.5 equiv.) is obtained. EPR spectroscopy indicates that, very likely, K^+^[dbcot^‒•^] (g = 2.00565) is formed in this reaction as well (Supplementary Scheme S1).

Finally, the oxidation of the dianion salts **K_2_trop** and **K_2_dbcot** with dry oxygen was investigated at low temperature. Remarkably, the oxidation of the paramagnetic **K_2_trop** gives the hydrocarbon C_15_H_12_ (suberene = tropH) as a major product (95%) and not as expected **Ktrop** or **trop_2_**, which is formed in only 5% yield ((f) in Scheme 3). On contrast, the reaction between oxygen and **K_2_dbcot**, which contains the diamagnetic and aromatic dbcot^2‒^ dianion, is very clean and gives the neutral hydrocarbon **dbcot** in almost quantitative yield ((g) in Scheme 3).

## 3. Materials and Methods

### 3.1. General Comments

**Scheme 4:**
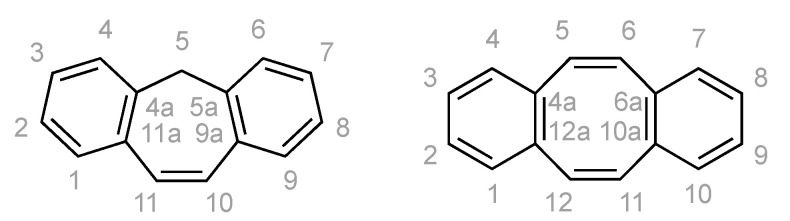
The assignment in the NMR spectra are based on these numbering schemes.

All experiments were performed under an argon atmosphere using standard Schlenk and vacuum-line techniques or in a MBraun inert-atmosphere drybox (argon atmosphere). All reagents were used as received from commercial suppliers unless otherwise stated. The following compounds were synthesized according to literature procedure: KC_8_ [50], dbcot [32] and [Rh_2_Cl_2_(cod)_2_] [51]. THF, DME, diethyl ether, toluene and *n*-hexane were purified using an Innovative Technologies PureSolv system and stored over 4 Å molecular sieves. THF-*d*_8_, C_6_D_6_ were distilled from sodium benzophenone ketyl. CDCl_3_ was distilled from CaH_2_. Solution NMR spectra were recorded on Bruker Avance 500, 400, 300, 250 and 200 MHz spectrometers. The chemical shifts (δ) are expressed in ppm relative to SiMe_4_ for ^1^H and ^13^C respectively. Coupling constants (*J*) are given in Hertz (Hz) as absolute values. The multiplicity of the signals is indicated as s, d, t, q, or m for singlets, doublets, triplets, quartets, or multiplets. If the data allowed it, the assignment was based on the IUPAC recommendations for fused polycyclic hydrocarbons (Scheme 4) [52]. Further abbreviations were used in the assignment: br for broadened signals, Ar for aromatic signals, quart for quaternary ^13^C signals.

EPR spectra were recorded by Dr. Reinhard Kissner on an X-band (9.50 GHz) Magnettech Miniscope 5000 EPR spectrometer with liquid nitrogen cooling. EPR spectra were simulated with Matlab R2016b, using EasySpin-5.2.11 package. UV/Vis spectra were recorded on a UV/Vis/NIR Lambda-19 spectrometer in a cell with a 2 mm path length. IR spectra were collected on a PerkinElmer Spectrum 2000 FT-IR-Raman spectrometer. Absorption bands are described as w, m or s for weak, medium or strong. Elemental analyses were performed by Peter Kälin in the Mikrolabor of the ETH Zürich. Melting points were determined with a Büchi melting-point apparatus and are not corrected. X-ray diffraction was performed at 100 K on an Oxford Xcalibur or Venture diffractometer with a CCD area detector and a molybdenum X-ray tube (0.71073 A). Using Olex^2^ [53], the structure was solved by direct methods (SHELXS [54] or SHELXT [55]), followed by least-squares refinement against full matrix (versus *F*^2^) with SHELXL [54]. All non-hydrogen atoms were refined anisotropically. The contribution of some hydrogen atoms, in their calculated positions, was included in the refinement using a riding model.

### 3.2. Synthesis

trop–Si(H)Me_2_ (**1a**): 5-Chloro-5*H*-dibenzo[*a*,*d*]cycloheptene (10.02 g, 44.20 mmol, 1.0 equivalent) and elementary lithium (0.71 g, 102.31 mmol, 2.3 equivalents) were reacted in dry tetrahydrofuran (40 mL) at a.T. overnight. The resulting dark red liquid was added dropwise to a solution of chlorodimethylsilane (5.4 mL, 4.60 g, 48.63 mmol, 1.1 equivalents) in dry tetrahydrofuran (40 mL) over an ice bath. The resulting yellow liquid was allowed to warm to a.T. and stirred overnight. The turbid suspension was concentrated under reduced pressure and subsequently distilled in high vacuum (only one fraction was observed from 90 to 115 °C). The obtained yellow liquid was placed in the fridge to give a white, crystalline solid after three days, which was triturated with dry diethyl ether (10.0 mL) and dried in high vacuum (8.328 g, 33.26 mmol, yield: 75.3%).

^1^H NMR (300 MHz, CDCl_3_, jb071.1.4) δ 7.27–7.09 (m, 6H, *H^Ar^*), 7.06 (dd, ^3^*J*_HH_ = 7.4 Hz, ^4^*J*_HH_ = 1.4 Hz, 2H, *H-4,6*), 6.74 (s, 2H, *H-10,11*), 4.18–4.07 (m, 1H, Si*H*), 3.49 (d, ^3^*J*_HH_ = 4.1 Hz, 1H, *H-5*), −0.09 (d, ^3^*J*_HH_ = 3.6 Hz, 6H, SiC*H*_3_) ppm. ^13^C NMR (75 MHz, CDCl_3_, jb071.1.4) δ 140.83 (s, 2C, *C^quart^*), 135.05 (s, 2C, *C^quart^*), 132.59 (s, 2C, *C-10,11*), 129.59 (s, 2C, *C^Ar^*), 129.13 (s, 2C, *C-4,6*), 128.96 (s 2C, *C^Ar^*), 125.71(s, 2C, *C^Ar^*), 47.60 (s, 1C, *C-5*), −3.71 (s, 2C, Si(*C*H_3_)_2_) ppm. ATR IR: λ^−1^: 3015 (w, *=C–H st*), 2955 (w, *–C–H st*), 2143 (m, *Si–H st*), 868 (s), 796 (s), 726 (s), 446 (s) cm^−1^. Elemental analysis: C (81.29%, calc.: 81.54%); H (7.37%, calc.: 7.24%).

trop–SiMe_3_ (**1b**): 5-Chloro-5*H*-dibenzo[*a*,*d*]cycloheptene (4.32 g, 19.06 mmol, 1.0 equivalent) and elementary lithium (0.33 g, 47.55 mmol, 2.5 equivalents) were reacted in dry tetrahydrofuran (30 mL) at a.T. overnight. The resulting deep red liquid was added dropwise to a colorless solution of trimethylsilyl chloride (3.2 mL, 2.74 g, 25.21 mmol, 1.3 equivalents) in dry tetrahydrofuran (35 mL). The reaction mixture was allowed to warm to a.T. and stirred overnight. The solvent of the orange solution was removed under reduced pressure to give an orange solid. The crude product was purified by sublimation (up to 150°C, HV) to give a yellow, crystalline solid (4.20 g, 15.88 mmol, yield: 83.3%).

^1^H NMR (300 MHz, C_6_D_6_, jb121.1.3) δ 7.08 (ddd, ^3^*J*_HH_ = 7.5 Hz, ^3^*J*_HH_ = 6.1 Hz, ^4^*J*_HH_ = 2.7 Hz, 2H, *H-3,7*), 7.06–6.92 (m, 4H, *H-1,2,8,9*), 6.86 (d, ^3^*J*_HH_ = 7.4 Hz, 2H, *H-4,6*), 6.48 (s, 2H, *H-10,11*), 3.41 (s, 1H, *H-5*), −0.06 (s, 9H, SiC*H*_3_) ppm. ^13^C NMR (75 MHz, C_6_D_6_, jb121.1.3) δ 140.91 (s, 2C, *C-4a,5a*), 135.49 (s, 2C, *C-9a,11a*), 132.86 (s, 2C, *C-10,11*), 129.95 (s, 2C, *C-1,9*), 129.73 (s, 2C, *C-4,6*), 129.07 (s, 2C, *C-3,7*), 125.71 (s, 2C, *C-2,8*), 50.24 (s, 1C, *C-5*), −0.39 (s, 3C, Si*C*H_3_) ppm. Elemental analysis: C (81.70%, calc.: 81.76%); H (7.74%, calc.: 7.62%).

**Litrop**: Method A: Lithium sand (0.348 g, 50.20 mmol, 2.3 equivalents) was suspended in dry tetrahydrofuran (10 mL) and placed in a cooling bath (propan-2-ol and dry ice). 5-Chloro-5*H*-dibenzo[*a*,*d*]cyclo-heptene (5.00 g, 22.06 mmol, 1.0 equivalent) was dissolved in dry tetrahydrofuran (30 mL). The resulting light yellow solution was added dropwise (during 25 min) to the brown suspension. The reaction mixture was allowed to warm to a.T. and a color change to dark red was observed under heat development. After two hours, the dark red liquid was concentrated to approximately 25 mL and filtered over a plug of celite. The resulting red solution was layered with dry *n*-hexane (71 mL) and stored in the freezer (−23 °C, 6 days). A first crop of green, crystalline needles was obtained (0.479 g, 1.26 mmol, yield: 2.5%; according to ^1^H NMR spectroscopy, the compound contained 2.5 equivalents of THF). The supernatant was layered with dry *n*-hexane (30 mL) and stored in the freezer (−23 °C, 1 day) and a second crop was obtained (7.448 g, 19.68 mmol,^1^ yield: 94.0%, overall yield: 95.0%).

Method B: (5*H*-Dibenzo[*a*,*d*]cycloheptene-5-yl)-dimethyl-silane (**1a**, 0.159 g, 0.64 mmol, 1.0 equivalent) was dissolved in dry 1,2-dimethoxyethane (2.0 mL). The resulting slightly yellow solution was added to a white suspension of lithium *tert*-butoxide (0.051 g, 0.64 mmol, 1.0 equivalent) in dry 1,2-dimethoxyethane (2.0 mL) (in a solvent with lower polarity (such as diethyl ether or tetrahydrofuran), a significantly lower yield was obtained). A swift color change to dark red was observed and the reaction mixture was stirred at a.T. (3 h). The dark red liquid was filtered over glass filter paper and celite, layered with dry *n*-hexane (10.0 mL) and stored in the freezer (−30 °C, overnight). The light yellow supernatant was discarded and the obtained green crystals were washed with dry *n*-hexane (3 × 1 mL) and dried under a stream of argon (0.1170 g, 0.35 mmol, yield: 55.2%; according to ^1^H NMR spectroscopy, the compound contained 1.5 equivalents of dme).

^1^H NMR (300 MHz, C_6_D_6_/THF-*d*_8_, jb070.1.6) δ 5.70 (td, ^3^*J*_HH_ = 7.5 Hz, ^4^*J*_HH_ = 1.6 Hz, 2H, *H-3,7*), 5.15 (td, ^3^*J*_HH_ = 7.1 Hz, ^4^*J*_HH_ = 1.3 Hz, 2H, *H-2,8*), 4.87 (dd, ^3^*J*_HH_ = 7.1 Hz, ^4^*J*_HH_ = 1.6 Hz, 2H, *H-1,9*), 4.59 (dd, ^3^*J*_HH_ = 7.9 Hz, ^4^*J*_HH_ = 1.3 Hz, 2H, *H-4,6*), 3.92 (s, 2H, *H-10,11*), 1.54 (s, 1H, *H-5*) ppm. ^1^H NMR (400 MHz, THF-*d_8_*, jb070.1.7) δ 4.80–4.73 (m, 2H, *H-3,7*), 4.12–4.05 (m, 2H, *H-2,8*), 3.73–3.66 (m, 2H, *H-1,9*), 3.48–3.42 (m, 2H, *H-4,6*), 2.64 (s, 2H, *H-10,11*), 0.50 (s, 1H, *H-5*) ppm. ^13^C NMR (101 MHz, THF-*d_8_*, jb070.1.7) δ 163.60 (s, 2C, *C-4a,5a*), 139.03 (s, 2C, *C-10,11*), 138.27 (s, 2C, *C-9a,11a*), 132.65 (s, 2C, *C-3,7*), 132.05 (s, 2C, *C-1,9*), 120.67 (s, 2C, *C-4,6*), 112.44 (s, 2C, *C-2,8*), 83.67 (s, 1C, *C-5*) ppm. UV/Vis (THF): λ_max_ 233 (ε: 1276(321) m^2^mol^−1^), 287 (ε: 2984(340) m^2^mol^−1^), 443 (ε: 631(102) m^2^mol^−1^), 513 (shoulder, ε: 411(65) m^2^mol^−1^) nm. ATR IR: λ^−1^: 2985 (w, *–C–H st*), 2926 (w, *–C–H st*), 1364 (s), 1068 (s), 912 (s), 724 (s), 436 (s) cm^−1^. Elemental Analysis for Litrop(dme)_1.7_: C (74.79%, calc.: 75.07%); H (7.78%, calc.: 7.95%). MP: 62 °C (decomp.).

**Natrop**: (5*H*-Dibenzo[*a*,*d*]cycloheptene-5-yl)-dimethyl-silane (**1a**, 2.005 g, 8.01 mmol, 1.0 equivalent) was dissolved in dry diethyl ether (30 mL). The resulting colorless solution was added to a white suspension of sodium *tert*-butoxide (0.769 g, 8.00 mmol, 1.0 equivalent) in dry diethyl ether (60 mL) over an ice bath. After 30 min, the reaction mixture was allowed to warm to a.T. and a dark red liquid was obtained. The solvent was removed under reduced pressure and dry 1,2-dimethoxyethane (30 mL) was added to the red residue. The liquid was filtered over a plug of celite, layered with dry *n*-hexane (80 mL) and stored in the freezer (−23°C, 2 days). The light yellow supernatant was discarded and the obtained dark green crystals were washed with dry *n*-hexane (2 × 10 mL) and briefly dried in vacuo (1.931 g, 6.16 mmol, yield: 77.0%; according to ^1^H NMR spectroscopy the compound contained 1.1 equivalents of dme).

^1^H NMR (300 MHz, THF-*d_8_*, jb075.3) δ 4.76 (ddd, ^3^*J*_HH_ = 7.8 Hz, ^3^*J*_HH_ = 7.1 Hz, ^4^*J*_HH_ = 1.6 Hz, 2H, *H-3,7*), 4.08 (td, ^3^*J*_HH_ = 7.1 Hz, ^4^*J*_HH_ = 1.2 Hz, 2H, *H-2,8*), 3.68 (dd, ^3^*J*_HH_ = 7.1 Hz, ^4^*J*_HH_ = 1.6 Hz, 2H, *H-1,9*), 3.43 (d, ^3^*J*_HH_ ≈ 6.9 Hz^§^, 2H, *H-4,6*), 2.60 (s, 2H, *H-10,11*), 0.56 (s, 1H, *H-5*) ppm. ^1^H NMR (400 MHz, C_6_D_6_/THF-*d_8_*, krastefa.50) δ 5.18 (td, ^3^*J*_HH_ = 7.5 Hz, ^4^*J*_HH_ = 1.6 Hz, 2H, *H-3,7*), 4.54 (td, ^3^*J*_HH_ = 7.1 Hz, ^4^*J*_HH_ = 1.3 Hz, 2H, *H-2,8*), 4.11 (dd, ^3^*J*_HH_ = 7.1 Hz, ^4^*J*_HH_ = 1.6 Hz, 2H, *H-1,9*), 3.93 (dd, ^3^*J*_HH_ = 7.9 Hz, ^3^*J*_HH_ = 1.4 Hz, 2H, *H-4,6*), 2.98 (s, 1H, *H-10,11*), 1.10 (s, 1H, *H-5*) ppm. ^13^C NMR (101 MHz, C_6_D_6_/THF-*d_8_*, krastefa.51) δ 161.95 (s, 2C, *C-4a,5a*), 138.79 (s, 2C, *C-10,11*), 136.39 (s, 2C, *C-9a,11a*), 133.16 (s, 2C, *C-3,7*), 132.57 (s, 2C, *C-1,9*), 120.12 (s, 2C, *C-4,6*), 113.22 (s, 2C, *C-2,8*), 81.56 (s, 1C, *C-5*) ppm. UV/Vis (THF): λ_max_ 285 (ε: 3074(349) m^2^mol^−1^), 459 (ε: 803(158) m^2^mol^−1^) nm. ATR IR: λ^−1^: 3039 (w, *=C–H st*), 3003 (w, *=C–H st*), 2978 (w, *–C–H st*), 2932 (w, *–C–H st*), 2913 (w, *–C–H st*), 2869 (w, *–C–H st*), 2823 (w, *–C–H st*), 1553 (s), 1443 (s), 1362 (s), 1118 (s), 1082 (s), 1028 (s), 734 (s) cm^−1^. Elemental Analysis for Natrop(dme)_1.1_: C (74.20%, calc.: 74.36%); H (6.92%, calc.: 7.08%). MP: 45 °C (decomp.).

^§^ The upper part of the multiplet lies underneath the signal of the solvent DME.

**Ktrop**: Method A: (5*H*-Dibenzo[*a*,*d*]cyclo-hepten-5-yl)-dimethyl-silane (**1a**, 1.500 g, 5.99 mmol, 1.0 equivalent) and potassium *tert*-butoxide (0.693 g, 6.18 mmol, 1.0 equivalent) were placed under argon. Dry tetrahydrofuran (20 mL) was added and a dark red liquid was obtained immediately. The reaction mixture was stirred at a.T. (10 hr) and filtered over a plug of celite. The dark red filtrate was layered with dry *n*-hexane (68 mL) and stored in the freezer (−23 °C, 4 days). A first crop of red crystals was isolated (0.624 g, 2.34 mmol, yield: 39.1%; according to ^1^H NMR spectroscopy the compound contained 0.5 equivalent of THF). The supernatant was layered with dry *n*-hexane (60 mL), stored in the freezer (−23 °C, 3 days) and a second crop was obtained (0.461 g, 2.00 mmol, yield: 32.4%, overall yield: 71.5%).

Method B: (5*H*-Dibenzo[*a*,*d*]cycloheptene-5-yl)-trimethyl-silane (**1b**, 4.909 g, 18.57 mmol, 1.0 equivalent) and potassium *tert*-butoxide (2.085 g, 18.58 mmol, 1.0 equivalent) were placed under argon. Dry tetrahydrofuran (35 mL) was added over an ice bath. The yellow solution was allowed to warm to a.T. and a gradual color change to dark red was observed. The reaction mixture was stirred overnight. The solvent was removed under reduced pressure. The red residue was triturated with dry diethyl ether (2 × 20 mL) and then washed with dry diethyl ether (40 mL). The resulting red solid was briefly dried in vacuo (3.552 g, 15.42 mmol, yield: 83.1%). The red solid (0.271 g, 1.18 mmol) was recrystallized in dry tetrahydrofuran (5.0 mL), filtered over glass filter paper and celite, layered with dry *n*-hexane (10.0 mL) and stored in the freezer (−30 °C, 2.5 days). The long, crystalline needles were washed with dry *n*-hexane (4 × 1.5 mL) and dried under a stream of argon (0.171 g, 0.74 mmol, yield of recrystallization: 63%).

^1^H NMR (300 MHz, THF-*d*_8_, jb073.1.1EA) δ 4.47 (ddd, ^3^*J*_HH_ = 7.9 Hz, ^3^*J*_HH_ = 7.1 Hz, ^4^*J*_HH_ = 1.6 Hz, 2H, *H-3,7*), 3.74 (td, ^3^*J*_HH_ = 7.1 Hz, ^4^*J*_HH_ = 1.2 Hz, 2H, *H-2,8*), 3.26 (dd, ^3^*J*_HH_ = 7.1 Hz, ^4^*J*_HH_ = 1.6 Hz, 2H, *H-1,9*), 2.97 (dd, ^3^*J*_HH_ = 8.0 Hz, ^4^*J*_HH_ = 1.3 Hz, 2H, *H-4,6*), 2.00 (s, 2H, H-10,11), 0.06 (s, 1H, H-5) ppm. ^1^H NMR (400 MHz, THF-*d*_8_, krastefa.11, T = 273 K) δ 4.40 (ddd, ^3^*J*_HH_ = 8.2 Hz, ^3^*J*_HH_ = 7.1 Hz, ^4^*J*_HH_ = 1.6 Hz, 2H, *H-3,7*), 3.65 (td, ^3^*J*_HH_ = 7.0 Hz, ^4^*J*_HH_ = 1.3 Hz, 2H, *H-2,8*), 3.18 (dd, ^3^*J*_HH_ = 7.1 Hz, ^4^*J*_HH_ = 1.6 Hz, 2H, *H-1,9*), 2.89 (dd, ^3^*J*_HH_ = 8.0 Hz, ^4^*J*_HH_ = 1.3 Hz, 2H, *H-4,6*), 1.91 (s, 2H, *H-11,10*), −0.01 (s, 1H, *H-5*) ppm. ^13^C NMR (101 MHz, THF-*d*_8_, krastefa.12, T = 273 K) δ 162.96 (s, 2C, *C-4a,5a*), 139.26 (s, 2C, *C-10,11*), 138.42 (s, 2C, *C-9a,11a*), 133.95 (s, 2C, *C-3,7*), 132.89 (s, 2C, *C-1,9*), 120.17 (s, 2C, *C-4,6*), 111.87 (s, 2C, *C-2,8*), 89.44 (s, 1C, *C-5*) ppm. UV/Vis (THF): λ_max_ 286 (ε: 3343(566) m^2^mol^−1^), 296 (shoulder, ε: 3086(595) m^2^mol^−1^), 478 (ε: 1025(277) m^2^mol^−1^) nm. ATR IR: λ^−1^: 3006 (w, *=C–H st*), 2985 (w, *–C–H st*), 2939 (w, *–C–H st*), 2924 (w, *–C–H st*), 1366 (m), 1121 (s), 1039 (m), 914 (m), 797 (m), 740 (s), 436 (m) cm^−1^. Elemental Analysis: C (78.25%, calc.: 78.21%); H (4.94%, calc.: 4.81%). MP: 240 °C (decomp.).

**K_2_trop**: **Ktrop** (0.461 g, 2.00 mmol, 1.0 equivalent) was dissolved in dry tetrahydrofuran (5.0 mL). The resulting dark red solution was added to a golden suspension of potassium graphite (0.355 g, 2.62 mmol, 1.3 equivalents) in dry tetrahydrofuran (5.0 mL) and stirred at a.T. (3 days). The dark red suspension was filtered over a plug of celite and the dark red filter cake was washed with dry tetrahydrofuran (3.5 mL). The dark red filtrate was split into two portions and each was layered with dry *n*-hexane (6.0 mL) and stored in the freezer (−30 °C, 3 days). The dark red, microcrystalline solid of both fractions was washed with dry *n*-hexane (2 × 2 mL each) and briefly dried in vacuo (0.372 g, 0.77 mmol, yield: 38.3%). The supernatant of both fractions was concentrated under reduced pressure and the dark residue was recrystallized again from dry tetrahydrofuran (3.0 mL) and dry *n*-hexane (6.0 mL) at −30 °C to give a second crop of dark red, microcrystalline solid (0.060 g, 0.12 mmol, yield: 6.2%, overall yield: 44.5%).

UV/Vis (THF): λ_max_ 286 (ε: 4098(190) m^2^mol^−1^), 297 (shoulder, ε: 3857(204) m^2^mol^−1^), 361 (ε: 1988(237) m^2^mol^−1^), 482 (ε: 1633(37) m^2^mol^−1^) nm. ATR IR: λ^−1^: 3020 (w, *=C–H st*), 2970 (w, *–C–H st*), 2868 (w, *–C–H st*), 1324 (m), 1015 (m), 736 (m), 686 (m), 433 (m) cm^−1^. Elemental Analysis for K_2_trop(thf)(*n*-hexane)_0.2_: C (67.95%, calc.: 67.62%); H (5.92%, calc.: 6.12%). MP: >250 °C.

**Na_2_dbcot**: Dibenzo[*a*,*e*]cyclooctatetraene (0.507 g, 2.48 mmol, 1.0 equivalent) was dissolved in dry tetrahydrofuran (10 mL). The colorless solution was added to a Schlenk tube with a sodium mirror (0.55 g, 23.92 mmol, 9.6 equivalents). A swift color change to dark red was observed. The reaction mixture was stirred at a.T. (1 day). The solvent was removed under reduced pressure. The purple residue was suspended in dry tetrahydrofuran (7 mL) and filtered over a plug of celite to give a dark red filtrate (a) and a dark red filter cake (b). (a) The dark red filtrate was layered with dry *n*-hexane (10 mL) and stored at a.T. (7 days). The obtained dark crystals were washed with dry *n*-hexane (2 mL) and dried under a stream of argon (0.145 g, 0.34 mmol, yield: 13.6%). (b) The filter cake was extracted with dry 1,2-dimethoxyethane (6 mL). The dark red filtrate was layered with dry *n*-hexane (8 mL) and stored in the freezer (−30 °C, 6 days). The obtained dark crystals were washed with dry *n*-hexane (2 × 2 mL) and dried under a stream of argon (0.256 g, 0.45 mmol, yield: 18.3%, overall yield: 31.9%).

^1^H NMR (300 MHz, THF-*d*_8_, jb105.1.2) δ 7.87 (dd, ^3^*J*_HH_ = 6.6 Hz, ^4^*J*_HH_ = 3.3 Hz, 4H, *H-1,4,7,10*), 7.17 (s, 4H, *H-5,6,11,12*), 6.21 (dd, ^3^*J*_HH_ = 6.6 Hz, ^4^*J*_HH_ = 3.4 Hz, 4H, *H-2,3,8,9*) ppm. ^13^C NMR (75 MHz, THF-*d*_8_, jb105.1.2) δ 135.26 (s, 4C, *C-1,4,7,10*), 108.90 (s, 4C, *C-2,3,8,9*), 107.43 (s, 4C, *C-4a,6a,10a,12a*), 93.37 (s, 4C, *C-5,6,11,12*) ppm. UV/Vis (THF): λ_max_ 238 (ε: 2523(2) m^2^mol^−1^), 261 (shoulder, ε: 1842(132) m^2^mol^−1^), 328 (ε: 5293(512) m^2^mol^−1^), 392 (ε: 853(87) m^2^mol^−1^), 514 (ε: 94(12) m^2^mol^−1^), 553 (ε: 106(13) m^2^mol^−1^), 597 (ε: 76(9) m^2^mol^−1^) nm.

**K_2_dbcot**: Dibenzo[*a*,*e*]cyclooctatetraene (0.301 g, 1.47 mmol, 1.0 equivalent) was dissolved in dry tetrahydrofuran (12 mL). Potassium graphite (0.526 g, 3.89 mmol, 2.6 equivalents) was added. The resulting dark red suspension was allowed to stir at a.T. (1 day). The reaction mixture was filtered over a plug of celite. The filter cake was washed with dry tetrahydrofuran (3 mL). The filtrate was split into two portions and each layered with dry *n*-hexane (8 mL) and stored in the freezer (−30 °C, 3 days). The obtained dark green crystals were washed with dry *n*-hexane (2 × 1 mL) and briefly dried in vacuo (0.450 g, 1.15 mmol, yield: 78.3%). The supernatant was concentrated under reduced pressure and the black residue was recrystallized in the same manner to give a second crop (0.047 g, 0.12 mmol, yield: 8.2%, overall yield: 86.6%).

^1^H NMR (300 MHz, THF-*d*_8_, jb085.1.1) δ 7.87 (dd, ^3^*J*_HH_ = 6.7 Hz, ^4^*J*_HH_ = 3.5 Hz, 4H, *H-1,4,7,10*), 7.17 (s, 4H, *H-5,6,11,12*), 6.19 (dd, ^3^*J*_HH_ = 6.7 Hz, ^4^*J*_HH_ = 3.3 Hz, 4H, *H-2,3,8,9*) ppm. ^13^C NMR (75 MHz, THF-*d*_8_, jb085.1.1) δ 135.72 (s, 4C, *C-1,4,7,10*), 109.36 (s, 4C, *C-4a,6a,10a,12a*), 108.91 (s, 4C, *C-2,3,8,9*), 95.78 (s, 4C, *C-5,6,11,12*) ppm. UV/Vis (THF): λ_max_ 235 (ε: 3997(184) m^2^mol^−1^), 261 (shoulder, ε: 1053(87) m^2^mol^−1^), 326 (ε: 1959(338) m^2^mol^−1^), 388 (ε: 334(53) m^2^mol^−1^), 502 (ε: 38(6) m^2^mol^−1^), 544 (ε: 42(7) m^2^mol^−1^), 587 (ε: 31(5) m^2^mol^−1^) nm. MP: >250 °C.

**[Rh**(**trop**)(**cod**)**]**: Cyclooctadiene rhodium(I) chloride dimer (0.050 g, 0.10 mmol, 1.0 equivalent) and **Ktrop** (0.047 g, 0.20 mmol, 2.0 equivalents) were stirred in dry toluene (6 mL) at a.T. (12 hr). The dark red liquid was filtered over a plug of celite, layered with dry *n*-hexane (6 mL) and stored in the freezer (−30 °C, 13 days). The obtained dark red crystals were washed with dry *n*-hexane (0.5 mL) and dried under a stream of argon (0.052 g, 0.13 mmol, yield: 63.0%).

^1^H NMR (400 MHz, C_6_D_6_, jb083.8A) δ 7.01–6.88 (m, 6H, *H^Ar^*), 6.85 (dd, ^3^*J*_HH_ = 7.1 Hz, ^1^*J*_HH_ = 1.7 Hz, 2H, *H-4,6*), 5.16 (d, ^2^*J*_RhH_ = 1.2 Hz, 2H, *H-10,11*), 4.09 (s_br_, 2H, C*H*^COD^), 3.59 (s_br_, 2H, C*H*^COD^), 2.88 (s, 1H, *H-5*), 2.21 (s_br_, 4H, C*H_2_*^COD^), 1.76 (s_br_, 4H, C*H_2_*^COD^) ppm. ^13^C NMR (101 MHz, C_6_D_6_, jb083.8A) δ 137.43 (s, 2C, *C-4a,5a*), 132.63 (s, 2C, *C-9a,11a*), 128.06 (s, 2C, *C-1,9/3,7*^§^), 126.86 (s, 2C, *C-4,6*), 126.66 (s, 2C, *C-3,7/1,9*), 126.13 (s, 2C, *C-2,8*), 97.52 (d, ^1^*J*_RhC_ = 5.4 Hz, 2C, *C-10,11*), 91.23 (s, 2C, *C*H^COD^), 70.69 (s, 2C, *C*H^COD^), 55.84 (d, ^1^*J*_RhC_ = 13.2 Hz, 1C, *C-5*), 35.22 (s, 2C, *C*H_2_^COD^), 29.91 (s, 2C, *C*H_2_^COD^) ppm.

^§^ The peak is buried under the signal for C_6_D_6_.

^1^H NMR (500 MHz, THF-*d*_8_, jb083.8.2A, −40 °C) δ 7.10–6.99 (m, 6H, *H^Ar^*), 6.89–6.84 (m, 2H, *H-4,6*), 5.46 (s, 2H, *H-10,11*), 3.92 (m_br_, 2H, C*H*^COD^), 3.70 (m_br_, 2H, C*H*^COD^), 2.81 (s, 1H, *H-5*), 2.30 (m, 4H, C*H_2_*^COD^), 1.76 (m, 4H, C*H_2_*^COD^) ppm. ^13^C NMR (126 MHz, THF-*d*_8_, jb083.8.2A, −40 °C) δ 137.75 (s, 2C, *C-4a,5a*), 133.25 (s, 2C, *C-9a,11a*), 128.54 (s, 2C, *C-1,9/3,7*), 127.42 (s, 2C, *C-3,7/1,9*), 127.24 (s, 2C, *C-4,6*), 126.78 (s, 2C, *C-2,8*), 97.57 (d, ^1^*J*_RhC_ = 5.0 Hz, 2C, *C-10,11*), 90.61 (d, ^1^*J*_RhC_ = 6.9 Hz, 2C, *C*H^COD^), 70.35 (d, ^1^*J*_RhC_ = 15.6 Hz, 2C, *C*H^COD^), 56.45 (d, ^1^*J*_RhC_ = 13.0 Hz, 1C, *C-5*), 35.09 (s, 2C, *C*H_2_^COD^), 30.46 (s, 2C, *C*H_2_^COD^) ppm.

## 4. Conclusions

A desilylation reaction using silyl trop derivative which proceeds by addition of KO*t*Bu allowed the very clean synthesis of larger amounts of the salts of the trop anion, **trop^‒^**, which contain alkali metal counter cations M^+^ = Li, Na, K. With these at hand, an in-depth study on the electronic configuration of these conjugated hydrocarbons exclusively composed from *sp^2^*-valence electron hybridized carbon centers could be performed. As the Hückel counting rules imply, the 16π-electron configuration of trop^‒^ predicts this to be anti-aromatic. This is indeed the case as NMR data and as well as calculated NICS data clearly show. A number of contact ion pairs, **Mtrop**, could be structurally characterized by X-ray diffraction methods. In these, ethereal molecules complete the coordination sphere around the M^+^ cation. From tetrahydrofuran (THF), close ion pairs are obtained which show an intimate contact between cation and trop^‒^ anion. In these, the trop^‒^ anion is bent and its anti-aromaticity lowered. On the contrast, crystallization from solvents, which have a higher solvation energy for alkali cations, lead to separated ion pairs in which the trop^‒^ anion is flatter and consequently, more anti-aromatic. Moreover, Li^+^ shows a higher tendency to form contact ion pairs than the larger potassium cation with a significantly reduced charge density. This culminates in the isolation of [K(thf)_2_][trop]_∞_, which forms a one-dimensional coordination polymer in the solid state with long K^+^ trop^‒^ distances. This compound contains a flat trop^‒^ anion which shows very little variation of the C=C bond lengths, strongly shielded NMR signals, strongly positive NICS values and as such, fulfils all formal criteria requested for an anti-aromatic compound. In this light, the ease of synthesis from trop silyl ethers and alcoholates is somewhat surprising, indicating that **Mtrop** salts are remarkably stable. The trop^‒^ monoanion can be further reduced to give salts **M_2_trop** with a paramagnetic dianion radical, trop^2‒•^, which was isolated and fully characterized as **K_2_trop**. In this species, the anti-aromaticity is reduced compared to the mono-anion. In a reaction between a Rh(I) halide complex and trop^‒^, the complex **[Rh**(**trop**)(**cod**)**]** was prepared. Not unexpected, this pure organometallic 18 electron complex shows a very different structure in which the trop unit—especially its annulated benzo groups—is aromatized. This reaction also shows that trop anions may have potential as ligands for transition metal complexes and as a building block for main group element compounds. It remains to be seen in how far trop-type anions can be used as electron carriers related to the well-established use of arenes for that purpose [15].

## Figures and Tables

**Table 1:** Characteristic properties of the trop anions, the dbcot dianions and the reference compounds **trop_2_** and **dbcot**. Abbreviations: δ ^1^H and δ ^13^C: ^1^H and ^13^C NMR chemical shifts (ppm) respectively, bz: benzylic, ol: olefinic, ^1^*J*_HC_: coupling constant (Hz]) between the benzylic proton and carbon atom.

	δ ^1^H_ol_	δ ^13^C_ol_	δ ^1^H_bz_	δ ^13^C_bz_	^1^ *J*_HC_
**trop_2_**	6.90	131.43	4.90	52.18	
**Litrop**	2.64	139.03	0.50	83.67	139.8
**Natrop**	2.60	138.79	0.56	81.56	142.9
**Ktrop**	2.00	139.26	0.06	89.44	144.5
**[Rh(trop)(cod)]**	5.16	97.52	2.88	55.84	144.6
**dbcot** [32]	6.72	126.76			
**Li_2_dbcot** [28]	7.08				
**Na_2_dbcot**	7.17	93.37			
**K_2_dbcot**	7.17	95.78			

**Table 2:** Selected bond lengths (Å) for the trop anions (Figure 1). Abbreviation: Cnt: centroid.

	Li^thf^trop	Li^dme^trop ^a^		Na^dme^trop	K^thf^trop	K^18c6^trop	[Rh(trop)(cod)]
C1–C2	1.440(2)	1.430(15)	1.420(16)	1.4265(19)	1.428(3)	1.429(3)	1.470(2)
C2–C3	1.423(2)	1.422(14)	1.435(17)	1.4342(19)	1.433(2)	1.430(3)	1.435(2)
C3–C4	1.468(2)	1.40(2)	1.54(2)	1.455(3)	1.463(3)	1.461(3)	1.458(3)
C4–C5	1.338(3)	1.325(14)	1.324(14)	1.308(3)	1.332(3)	1.339(3)	1.380(3)
C5–C6	1.464(3)	1.47(3)	1.46(3)	1.414(3)	1.467(3)	1.464(3)	1.482(3)
C6–C7	1.423(2)	1.422(16)	1.412(17)	1.419(2)	1.430(3)	1.434(3)	1.398(2)
C7–C1	1.434(2)	1.422(13)	1.414(15)	1.419(2)	1.425(3)	1.431(3)	1.492(2)
C1–M1	2.279(3)	6.788(17)	7.91(2)	5.3528(15)	3.0977(17) ^b^	3.0213(18)	2.0912(16)
C4–M1	3.715(4)	7.63(2)	7.50(2)	5.4943(17)	3.4072(19)	4.937(3)	2.2397(17)
C5–M1	3.783(3)	8.35(2)	6.64(2)	5.5599(19)	3.346(2)	4.694(2)	2.4044(18)
M1–Cnt1		7.282(17)	7.34(2)	5.2530(15)	2.8223(4) ^c^	3.8117(18)	2.2600(16)
M1–Cnt2					2.9708(4) ^d^	3.303(2)	2.2186(18)
M1–Cnt3							1.9682(19)
M1–Cnt4							2.1139(19)

^a^ The asymmetric unit contains two distorted trop moieties: a and b. ^b^ Distance to benzylic C of neighboring trop anion: C1′–K1: 3.0171(17) (see **K^thf^trop** in Figure 1 for clarification). ^c^ Centroid of C1, C2, C5, C6, C7. ^d^ Allylic centroid of C1′, C2′ and C8′.

**Table 3:** Data from solid-state structures (Figure 1, Figure 2, and Figure 4). For comparison, **trop_2_**, **trop^+^**, and **Li_2_dbcot** are listed as well. Abbreviations: $\bar{C=C}$: average bond length in the central ring (Å), $\bar{\Delta C=C}$: average bond length difference in the central ring (Å), $\theta_{1}$ and $\theta_{2}$: dihedral angles in the trop scaffold (°), $\varphi_{1}$ and $\varphi_{2}$: dihedral angles in the **dbcot** scaffold (°), **K^18c6^trop**: **Ktrop** with 18-crown-6.

	Central Ring		Trop Angles		Dbcot Angles	
Compound	$\bar{C=C}$	$\bar{\Delta C=C}$	$\theta_{1}$	$\theta_{2}$	$\varphi_{1}$	$\varphi_{2}$
**trop_2_** [42]	1.4451(7)	0.0802(10)	49.37(12)	22.46(9)		
**trop^+^** [45],^**a**^	1.4134(11)	0.0283(15)	1.6(2)	3.44(14)		
**Li^thf^trop**	1.4271(9)	0.0537(12)	24.16(19)	14.13(14)		
**Li^dme^trop**	1.421(10)	0.060(14)	6(4)	2.9(25)		
**Natrop**	1.4108(9)	0.0420(13)	6.49(19)	4.66(12)		
**K^thf^trop**	1.4253(11)	0.0491(15)	1.7(2)	2.37(14)		
**K^18c6^trop**	1.4269(11)	0.0449(16)	12.4(3)	12.35(17)		
**K_2_trop**	1.4346(10)	0.0337(14)	1.3(2)	2.30(13)		
**[Rh(trop)(cod)]**	1.4450(9)	0.0626(13)	55.49(18)	32.29(13)		
**Li_2_dbcot** [31]	1.421(2)	0.024(2)			0 ^b,c^	
**Na_2_dbcot**	1.4281(5)	0.0234(7)			5.38(3)	4.88(3)
**K_2_dbcot**	1.4279(7)	0.0263(10)			1.40(12) ^b^	

^a^ The counter ion is tetrakis(3,5-bis(trifluoromethyl)phenyl)borate. ^b^ The respective point group leads to pairwise identical dihedral angles. ^c^ The localization of dbcot on a crystallographic mirror plane leads to the perfect planarity [31].

**Table 4:** Selected bond lengths (Å) for ion pairs containing the trop^2‒^ and dbcot^2‒^ dianions (Figure 2). Abbreviation: Cnt: centroid.

	K_2_trop	Na_2_dbcot	K_2_dbcot ^a,b^	
C1–C2	1.426(3)	1.4121(15)	1.409(2)	1.409(2)
C2–C3	1.463(2)	1.4254(14)	1.426(2)	1.418(2)
C3–C4	1.434(3)	1.4555(14)	1.460(2)	1.463(2)
C4–C5	1.385(3)	1.4223(14)		
C5–C6	1.440(3)	1.4094(15)		
C6–C7	1.466(2)	1.4209(14)		
C7–C8		1.4596(14)		
C4–C1			1.420(2)	1.418(2)
C7–C1	1.428(2)			
C8–C1		1.4199(14)		
C1–M1	3.2455(18)	2.6792(11)	3.0482(15)	3.0905(14)
C2–M1		2.7258(11)	3.0093(15)	3.0728(14)
C4–M1	3.2607(18)			
C5–M1	3.1092(17)	2.6971(11)		
C6–M1		2.8896(11)		
C1–M2	3.0902(17)	2.7507(11)		
C2–M2		2.6557(11)		
C4–M2	3.3042(18)			
C5–M2	3.1789(18)	2.9216(11)		
C6–M2		2.7689(11)		
M1–Cnt1	2.7876(18)	2.0924(11)	2.4669(15)	2.5385(14)
M2–Cnt1	2.7495(17)	2.1348(11)		
K1-Cnt1-K2	166.507(12)	174.05(4)	180.00(5)	180.00(4)

^a^ Two chemically identical, but crystallographically unique compounds are in the asymmetric unit. ^b^ The space group P2_1_/n leads to pairwise identical C–C bond lengths.

**Table 5:** Calculated isotropic NICS(0) and NICS(1) values for **trop^+^**, **trop^‒^**, model compounds **Mtrop^m^** (M = Li, Na, K) and **K_2_trop^m^**, **[Rh**(**trop**)(**cod**)**]** and cyclobutadiene (CBD). The model compounds do not contain solvent molecules attached to the alkali cations. Computational details: PBE [38] 6-311+G(df, pd) [39] in a continuum solvation model (THF). Abbreviations: *θ*_1_ and *θ*_2_: dihedral angles of the optimized structures (°), CBD: 1,3-cyclobutadiene.

		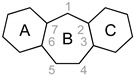			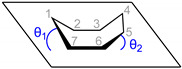	
		A	B	C	$\theta_{1}$	$\theta_{2}$
**trop^+^**	NICS(0)	−8.87	−4.71	−8.87	0.00	0.00
	NICS(1)	−11.76	−7.56	−11.76		
**trop^–^**	NICS(0)	21.05	45.00	21.02	0.01	0.01
	NICS(1)	15.12	34.54	15.10		
**Litrop^m^**	NICS(0)	6.41	25.23	6.66	26.19	13.54
	NICS(1) ^a^	3.85	18.82	3.89		
	NICS(1) ^b^	0.95	17.36	1.67		
**Natrop^m^**	NICS(0)	10.23	30.78	10.23	19.99	11.00
	NICS(1) ^a^	7.00	23.32	7.00		
	NICS(1) ^b^	4.40	22.04	4.41		
**Ktrop^m^**	NICS(0)	17.02	42.39	17.02	7.41	1.77
	NICS(1) ^a^	12.03	32.38	12.02		
	NICS(1) ^b^	11.18	32.86	11.18		
**[Rh(trop)(cod)]**	NICS(0)	−6.04	−4.33	−5.79	58.20	34.54
	NICS(1) ^a^	−7.41	−1.38	−7.90		
	NICS(1) ^b^	−8.47	−26.89	−8.53		
**K2trop^m^**	NICS(0)	4.77	12.87	4.77	0.02	0.02
	NICS(1)	1.93	8.78	1.93		
**CBD**	NICS(0)		27.49			
	NICS(1)		18.03			

^a^ NICS(1) opposite to the metal center. ^b^ NICS(1) on the same side as the metal center. CBD = cyclobutadiene, C_4_H_4_.

**Table 6:** Calculated isotropic NICS(0) and NICS(1) values (ppm) for dianionic dbcot compounds. Computational details: PBE [38] 6-311+G(df, pd) [39] in a continuum solvation model (THF). Abbreviations: $\varphi_{1}$ and $\varphi_{2}$: dihedral angles of the optimized structures (°).

		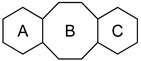			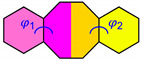	
		A	B	C	$\varphi_{1}$	$\varphi_{2}$
**dbcot^2-^**	NICS(0)	−6.60	−17.20	−6.60	0.07	0.07
	NICS(1)	−7.39	−15.13	−7.39		
**Li_2_dbcot^m^**	NICS(0)	−4.42	−15.27	−6.76	3.73	2.50
	NICS(1)	−6.51	−13.49	−8.45		
	NICS(1)	−5.31	−15.04	−7.77		
**Na_2_dbcot^m^**	NICS(0)	−5.59	−14.57	−5.59	0.03	0.05
	NICS(1)	−7.06	−14.15	−7.06		
**K_2_dbcot^m^**	NICS(0)	−5.41	−17.48	−5.42	0.03	0.04
	NICS(1)	−6.93	−16.96	−6.94		
**Benzene**	NICS(0)		−7.68			
	NICS(1)		−10.09			

## Supplementary Materials

The following are available online: 1. DFT-Calculations; Table S1: Selected bond lengths [Å] for the calculated trop mono- and dianions; Table S2: Key parameters for the determination of the number of electrons; Figure S1: Bottom: Recorded (red) vs. calculated (blue) spectrum of **Litrop** in THF; Figure S2: Bottom: Recorded (red) vs. calculated (blue) spectrum of **Natrop** in THF; Figure S3: Bottom: Recorded (red) vs. calculated (blue) spectrum of **Ktrop** in THF; Figure S4: Stacked spectra of **Litrop**, **Natrop** and **Ktrop** in THF; Figure S5: Bottom: Recorded (red) vs. calculated (blue) spectrum of **K_2_trop** in THF; Figure S6: Recorded (red) vs. calculated (blue) spectrum of **Na_2_dbcot** in THF; Figure S7: Recorded (red) vs. calculated (blue) spectrum of **K_2_dbcot** in THF; 2. Electrochemical data for **Ktrop** and **trop_2_** on GC electrode; Figure S8: Scan rate dependence of the 1st reduction process for **Ktrop** and trop_2_ in 0.1 M TBAPF_6_ solution in THF on glassy carbon electrode; Figure S10: Overlay of the I_p_ currents vs. scan rate^1/2^ plot of **Ktrop**^2-/3−^ and **trop_2_**^1-/2-^ redox processes with theoretical 1e^‒^ and 2e^−^ plot slopes; Figure S9: Overlay of the successive CV scans for 1 mM of **Ktrop** solution in THF (0.1 M TBAPF_6_) at 100 mVs^−1^; Figure S11: I vs. t^−1/2^ plot of the chronoamperometry data obtained from a 10 mM **trop_2_** solution at −3.07 V vs. Fc/Fc^+^ and a 10 mM ferrocene solution at 0.23 V vs. Fc/Fc^+^ in 0.1 M TBAPF_6_ in THF; Figure S12: Overlay of linear sweep voltammograms of a 10 mM **trop_2_** solution and a 10 mM ferrocene in a 0.1 M TBAPF_6_ solution in THF obtained in stationary regime at a carbon microelectrode (5 mVs^‒1^ scan rate); Figure S13: Overlay of the CVs recorded for **Ktrop**, **trop_2_**, and **trop_2_** in presence of 10 mM KPF_6_; Figure S14: Overlay of the cyclic voltammograms at various scan rates with 1 mM **trop_2_** in 0.1 M TBAPF_6_ solution in THF on glassy carbon electrode; Scheme S1: Selected interconversion reactions between the **trop** and the **dbcot** scaffold.
