# Supplementary material for: Synthesis and Characterization of Ion Pairs between Alkaline Metal Ions and Anionic Anti-Aromatic and Aromatic Hydrocarbons with π-Conjugated Central Seven- and Eight-Membered Rings
Source: Molecules. 2020 Oct 15;25(20):4742. doi: 10.3390/molecules25204742 (PMC7594067; doi:10.3390/molecules25204742)
Supplement: Supplementary file 1 [file molecules-25-04742-s001.pdf]

molecules

Supporting Information

Synthesis and Characterization of Ion Pairs between Alkaline Metal Ions and Anionic Anti-Aromatic and Aromatic Hydrocarbons with π-Conjugated Central Seven- and Eight-Membered Rings

Jan Bloch [1], Stefan Kradolfer [1], Thomas L. Gianetti [2], Detlev Ostendorf [1], Subal Dey [1], Victor Mougel [1] and Hansjörg Grützmacher [1,*]

[1] Department of Chemistry and Applied Biosciences, ETH Zürich, 8093 Zürich, Switzerland; bloch@inorg.chem.ethz.ch (J.B.); krastefa@inorg.chem.ethz.ch (S.K.); detos@gmx.de (D.O.); deys@ethz.ch (S.D.); mougel@inorg.chem.ethz.ch (V.M.)

[2] Department of Chemistry & Biochemistry, 1306 E. University Blvd., Tucson, AZ 85719, USA; tgianetti@email.arizona.edu

* Correspondence: hgruetzmacher@ethz.ch

1. DFT-Calculations

All structures and spectra were calculated using the Gaussian09 suite of programs [1].

Geometry optimizations were performed using the PBE density functional [2, 3] and verified by vibrational analysis. The influence of the solvent was taking in consideration as polarizable continuum solvent model. The basis set 6-311+g(df,pd) [4] was used for optimization and frequency calculations. The UV/Vis spectra were computed with TD-DFT using the same method and basis set as for the geometry optimizations.

Optimized X,Y,Z Cartesian coordinates [Å] used for NICS and UV/Vis calculations

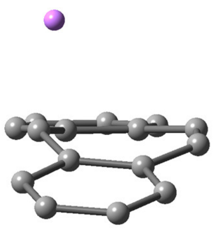

Litrop

C	-1.353416	-1.840325	-1.653584
C	-0.006107	-1.894405	-1.802844
C	1.023695	-0.85422	-1.72643
C	-2.256169	-0.723787	-1.360661
C	0.799823	0.438051	-1.137521
C	-1.805293	0.54406	-0.851084
C	-0.435684	0.888871	-0.528727
H	-1.877808	-2.788509	-1.823726
H	0.391633	-2.879431	-2.075564
H	-0.35511	1.927597	-0.176332
C	1.921832	1.313144	-1.112823
H	1.783302	2.308926	-0.680603
C	2.296743	-1.186079	-2.219005

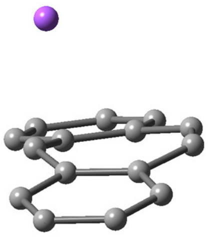

Natrop

C	-1.886485	0.250798	-0.678542
C	-1.886485	0.250798	0.678542
C	-0.847717	-0.082692	1.657752
C	-0.847717	-0.082692	-1.657752
C	0.54219	-0.24851	1.313688
C	0.54219	-0.24851	-1.313688
C	1.117214	-0.113729	0
H	-2.846616	0.511293	-1.140606
H	-2.846616	0.511293	1.140606
H	2.199987	-0.297627	0
C	1.418592	-0.529734	2.403865
H	2.480459	-0.67099	2.18017

H	2.433038	-2.182466	-2.6513
C	3.384761	-0.303761	-2.17282
H	4.359255	-0.596577	-2.566886
C	3.1788	0.957793	-1.604975
H	3.999863	1.677707	-1.548166
C	-3.629586	-0.952332	-1.548171
H	-3.936369	-1.932278	-1.927503
C	-2.823635	1.503156	-0.582582
H	-2.515014	2.482208	-0.203258
C	-4.18249	1.248631	-0.772603
H	-4.911626	2.029059	-0.538156
C	-4.608121	0.009991	-1.264448
H	-5.666185	-0.204996	-1.422155
Li	-0.409333	-0.161328	1.416072

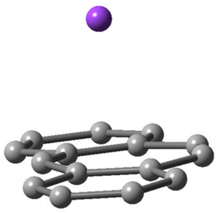

Ktrop

C	-1.764775	0.601534	0.67895
C	-1.764775	0.601534	-0.67895
C	-0.684057	0.507293	-1.667982
C	-0.684057	0.507293	1.667982
C	0.708803	0.34234	-1.316363
C	0.708803	0.34234	1.316363
C	1.252192	0.205216	0
H	-2.755957	0.699985	1.137352
H	-2.755957	0.699985	-1.137352
H	2.341621	0.082058	0
C	1.612848	0.270406	-2.42256
H	2.677769	0.155122	-2.199807
C	-1.062943	0.579147	-3.017958
H	-2.127418	0.707616	-3.239395
C	-0.147474	0.501428	-4.078327
H	-0.481594	0.565369	-5.114714
C	1.205855	0.345979	-3.752934
H	1.958382	0.285717	-4.544241
C	-1.062943	0.579147	3.017958
H	-2.127418	0.707616	3.239395
C	1.612848	0.270406	2.42256

C	-1.253503	-0.190802	2.997251
H	-2.316221	-0.050043	3.219537
C	-0.363866	-0.462442	4.047486
H	-0.718706	-0.54034	5.076276
C	0.988316	-0.627817	3.726919
H	1.71867	-0.841808	4.512288
C	-1.253503	-0.190802	-2.997251
H	-2.316221	-0.050043	-3.219537
C	1.418592	-0.529734	-2.403865
H	2.480459	-0.67099	-2.18017
C	0.988316	-0.627817	-3.726919
H	1.71867	-0.841808	-4.512288
C	-0.363866	-0.462442	-4.047486
H	-0.718706	-0.54034	-5.076276
Na	1.026476	2.441652	0

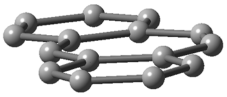

trop⁻

C	-1.838655	-0.000184	-0.678138
C	-1.838655	-0.000184	0.678138
C	-0.756293	-0.000108	1.666433
C	-0.756293	-0.000108	-1.666433
C	0.64679	-0.000202	1.312323
C	0.64679	-0.000202	-1.312323
C	1.199717	-0.000296	0
H	-2.835377	-0.000178	-1.137303
H	-2.835377	-0.000178	1.137303
H	2.297052	-0.000273	0
C	1.553269	-0.000046	2.421838
H	2.624861	-0.00006	2.198797
C	-1.13824	0.000109	3.014958
H	-2.21117	0.000269	3.236223
C	-0.219883	0.000278	4.080607
H	-0.559919	0.000584	5.117517
C	1.142255	0.000164	3.753429
H	1.898468	0.000336	4.544503
C	-1.13824	0.000109	-3.014958
H	-2.21117	0.000269	-3.236223
C	1.553269	-0.000046	-2.421838
H	2.624861	-0.00006	-2.198797
C	1.142255	0.000164	-3.753429

H	2.677769	0.155122	2.199807
C	1.205855	0.345979	3.752934
H	1.958382	0.285717	4.544241
C	-0.147474	0.501428	4.078327
H	-0.481594	0.565369	5.114714
K	-0.358749	-2.3115	0

H	1.898468	0.000336	-4.544503
C	-0.219883	0.000278	-4.080607
H	-0.559919	0.000584	-5.117517

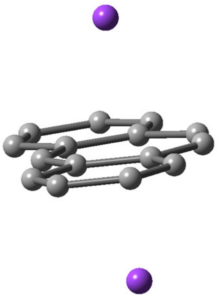

K_2trop

C	-0.000491	-1.755354	0.702334
C	-0.000491	-1.755354	-0.702334
C	-0.000093	-0.687601	-1.676382
C	-0.000093	-0.687601	1.676382
C	-0.000009	0.740166	-1.327649
C	-0.000009	0.740166	1.327649
C	-0.000142	1.291399	0
H	-0.000477	-2.755966	1.151947
H	-0.000477	-2.755966	-1.151947
H	0.000428	2.3893	0
C	0.000172	1.651203	-2.429509
H	0.000126	2.72093	-2.191095
C	0.000198	-1.038934	-3.054959
H	0.000212	-2.108784	-3.29474
C	0.000456	-0.11153	-4.096575
H	0.000741	-0.444878	-5.13683
C	0.0004	1.263146	-3.768978
H	0.000488	2.023989	-4.554145
C	0.000198	-1.038934	3.054959
H	0.000212	-2.108784	3.29474
C	0.000172	1.651203	2.429509
H	0.000126	2.72093	2.191095
C	0.0004	1.263146	3.768978
H	0.000488	2.023989	4.554145
C	0.000456	-0.11153	4.096575
H	0.000741	-0.444878	5.13683
K	2.563127	-0.256595	0
K	-2.56362	-0.256113	0

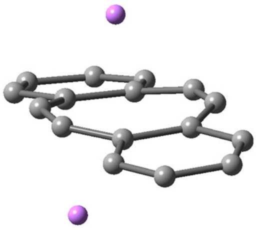

Li_2dbcot

C	1.837433	-0.707757	0.062148
C	1.828402	0.754987	0.050272
C	-1.791159	-0.702325	0.080154
C	0.725652	1.651341	0.157775
C	-1.792092	0.765523	-0.020474
C	-0.68614	1.664498	0.110553
H	1.091472	2.682053	0.244442
H	-1.051434	2.697739	0.150846
C	0.743979	-1.605426	0.235636
H	1.103267	-2.632841	0.37048
C	-0.684064	-1.588961	0.293217
H	-1.05331	-2.605999	0.471075
C	3.111204	1.404234	-0.052852
H	3.106121	2.499498	-0.049237
C	3.128697	-1.347438	-0.045221
H	3.136971	-2.442729	-0.026462
C	4.316099	0.748748	-0.163968
H	5.247596	1.313149	-0.249392
C	4.323137	-0.676645	-0.159555
H	5.261635	-1.231107	-0.236748
C	-3.086167	-1.349243	0.033324
H	-3.091232	-2.440228	0.129529
C	-3.077515	1.399653	-0.195351
H	-3.073613	2.491516	-0.278461
C	-4.278503	-0.691898	-0.139991
H	-5.214781	-1.253396	-0.181165
C	-4.275541	0.73108	-0.267206
H	-5.208187	1.28091	-0.411234
Li	-0.79265	0.214749	1.913356
Li	0.020968	-1.23501	-1.848167

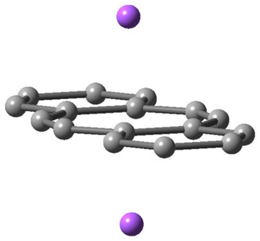

Na₂dbcot

C	-1.819259	-0.7348	0.000629
C	-1.819136	0.734483	0.000108
C	1.818887	-0.734722	-0.000028
C	-0.710745	1.635181	-0.000412
C	1.818992	0.734574	0.000017
C	0.710621	1.635291	-0.00045
H	-1.072489	2.669927	-0.000678
H	1.072253	2.670074	-0.00057
C	-0.710875	-1.635479	0.000953
H	-1.072507	-2.670267	0.001285
C	0.710473	-1.635394	0.000539
H	1.072193	-2.670152	0.000548
C	-3.113363	1.376612	0.000329
H	-3.113655	2.471989	0.000395
C	-3.113513	-1.376824	0.000089
H	-3.113866	-2.4722	-0.000048
C	-4.317365	0.713778	0.000221
H	-5.255695	1.273399	0.000312
C	-4.317456	-0.713875	-0.00015
H	-5.255851	-1.273384	-0.000585
C	3.113111	-1.376853	-0.001165
H	3.113385	-2.472228	-0.001894
C	3.113278	1.376587	0.000631
H	3.113646	2.471962	0.001297
C	4.317117	-0.714029	-0.001064
H	5.255444	-1.273653	-0.001844
C	4.317206	0.713628	0.00022
H	5.255604	1.273135	0.000725
Na	-0.000665	0.000649	-2.104055
Na	0.001911	0.000482	2.103897

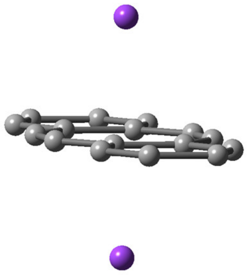

K₂dbcot

C	1.810646	-0.000019	-0.735314
C	1.810815	0.000381	0.735647
C	-1.810316	-0.000468	-0.735419
C	0.70881	0.000549	1.645476
C	-1.810165	0.00014	0.735561
C	-0.708087	0.000843	1.645313
H	1.076374	0.001157	2.678765
H	-1.075832	0.001264	2.678546
C	0.708583	-0.000986	-1.645123
H	1.076327	-0.001428	-2.678353
C	-0.708272	-0.001268	-1.645239
H	-1.075917	-0.001852	-2.678503
C	3.107491	0.000014	1.375278
H	3.108535	-0.000219	2.470727
C	3.107276	0.000478	-1.375084
H	3.10825	0.000555	-2.470535
C	4.312139	0.000174	0.713826
H	5.250185	-0.000026	1.274101
C	4.312015	0.000643	-0.713795
H	5.249967	0.000996	-1.274228
C	-3.106995	-0.000032	-1.375051
H	-3.108043	-0.000186	-2.4705
C	-3.106781	0.000421	1.375325
H	-3.107746	0.000654	2.470779
C	-4.311638	0.000456	-0.713598
H	-5.24969	0.000774	-1.27386
C	-4.311513	0.000525	0.714035
H	-5.249464	0.000742	1.274468
K	-0.000734	2.42897	-0.000899
K	-0.000687	-2.429682	0.000244

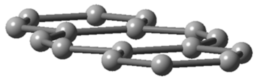

dbcot^{2-}

C	0.252081	0.781852	-0.561192
C	1.695365	0.607487	-0.815219
C	0.757941	4.332797	-0.119367
C	2.731438	1.582385	-0.83664
C	2.201224	4.158431	-0.373393
C	2.928931	2.970141	-0.662639
H	3.693529	1.098052	-1.061054
H	3.994343	3.209985	-0.796244
C	-0.475623	1.970141	-0.271935
H	-1.541033	1.730294	-0.138315
C	-0.278131	3.357897	-0.097935
H	-1.240219	3.842228	0.126494
C	2.132448	-0.73861	-1.083736
H	3.204954	-0.869875	-1.275131
C	-0.549011	-0.4144	-0.609283
H	-1.622013	-0.285608	-0.418959
C	1.318698	-1.854733	-1.119605
H	1.739966	-2.841405	-1.333664
C	-0.077682	-1.686349	-0.872188
H	-0.761429	-2.540132	-0.888142
C	0.320854	5.678897	0.149128
H	-0.751652	5.810161	0.340522
C	3.002314	5.354686	-0.32532
H	4.075316	5.225894	-0.515641
C	1.134602	6.795022	0.18498
H	0.713331	7.781696	0.399024
C	2.530982	6.626638	-0.062434
H	3.214727	7.480423	-0.046493

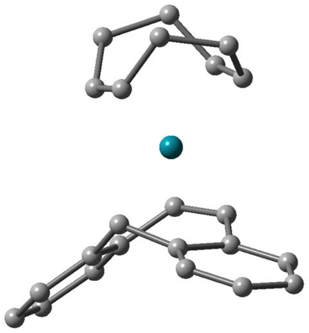

[Rh(trop)(cod)]

Rh	0.654429	-0.176032	0.071032
C	-0.842897	1.666316	-0.531095
C	-1.056212	0.302055	-1.059763
H	-0.932575	0.259781	-2.152198
C	-2.283012	-0.430772	-0.606159
C	-2.275726	-0.926702	0.710428
C	-0.629958	1.819571	0.888869
C	-3.383809	-0.675447	-1.431526
H	-3.382102	-0.300712	-2.458765
C	-0.542668	0.644783	1.756058
H	0.007353	0.80525	2.689576
C	-1.098507	-0.630576	1.555161
H	-0.903418	-1.349888	2.357303
C	-4.468282	-1.434902	-0.963079
H	-5.310522	-1.647262	-1.625109
C	-0.428443	4.056897	-0.830045
H	-0.348136	4.923807	-1.489238
C	-0.713825	2.805133	-1.360721
H	-0.855044	2.683192	-2.437337
C	-4.45474	-1.937674	0.339186
H	-5.287857	-2.542449	0.703525
C	-0.309125	3.10397	1.393779
H	-0.126704	3.215258	2.465512
C	-3.360086	-1.679419	1.177638
H	-3.343629	-2.078313	2.195466
C	-0.230881	4.207572	0.555524
H	-0.002017	5.191331	0.970167
C	1.781246	-0.70976	-1.640653
H	1.30796	-0.267584	-2.525315
C	1.134917	-1.86864	-1.103525
H	0.232329	-2.214284	-1.621607
C	2.673452	0.216559	0.887573
H	2.558089	1.069883	1.566251

C	2.265182	-1.037161	1.35756
H	1.831415	-1.075112	2.361902
C	3.278029	-0.428398	-1.512126
H	3.835499	-1.377515	-1.474574
H	3.625375	0.086007	-2.421248
C	2.758351	-2.364165	0.803979
H	2.843321	-3.08745	1.629342
H	3.774807	-2.241629	0.401203
C	3.608697	0.44799	-0.284347
H	4.660811	0.297915	0.027546
H	3.528399	1.508107	-0.571152
C	1.817251	-2.94014	-0.278997
H	2.373042	-3.638708	-0.934802
H	1.030561	-3.540717	0.20481

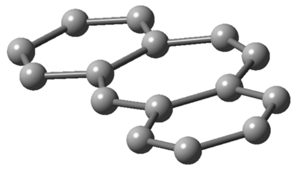

trop$^+$

C	0.000000	0.000000	1.583107
C	1.627994	0.000000	-0.377101
C	-1.627994	-0.000021	-0.377101
C	-0.687927	0.000016	-1.447194
C	0.687927	0.000025	-1.447194
C	1.296260	-0.000001	1.032743
C	-1.296260	-0.000018	1.032743
H	-1.145577	0.000033	-2.440206
H	1.145577	0.000048	-2.440206
H	0.000000	0.000008	2.677839
C	2.364075	-0.000002	1.984078
H	2.108982	0.000007	3.045133
C	2.999244	-0.000016	-0.735881
H	3.258352	-0.000018	-1.795774
C	4.003030	-0.000029	0.216291
H	5.047504	-0.000040	-0.099081
C	3.684487	-0.000016	1.592103
H	4.479715	-0.000016	2.338017
C	-2.999244	-0.000055	-0.735881
H	-3.258352	-0.000061	-1.795774
C	-2.364075	-0.000033	1.984078
H	-2.108982	-0.000020	3.045133
C	-3.684487	-0.000064	1.592103
H	-4.479715	-0.000075	2.338017
C	-4.003030	-0.000081	0.216291
H	-5.047504	-0.000107	-0.099081

Table S1. Selected bond lengths [Å] for the calculated trop mono- and dianions.

Compound	M–C5
**Litrop**	2.210
**Natrop**	2.557
**Ktrop**	2.988
**K_2trop**	2.994
**[Rh(trop)(cod)]**	2.106

**Excited state**	**1**	**2**	**4**
**λ/nm**	**933.19**	**476.11**	**443.32**
**Oscillator strength**	**0.0018**	**0.0137**	**0.1511**
	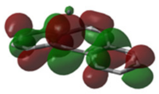	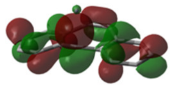	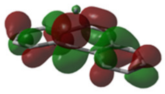
involved NTOs (iso value = 0.03)	52 (HOMO)	52 (HOMO)	52 (HOMO)
	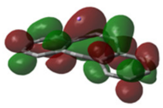	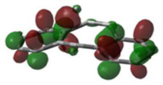	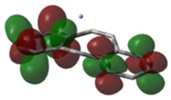
	53 (LUMO)	54 (LUMO+1)	56 (LUMO+3)

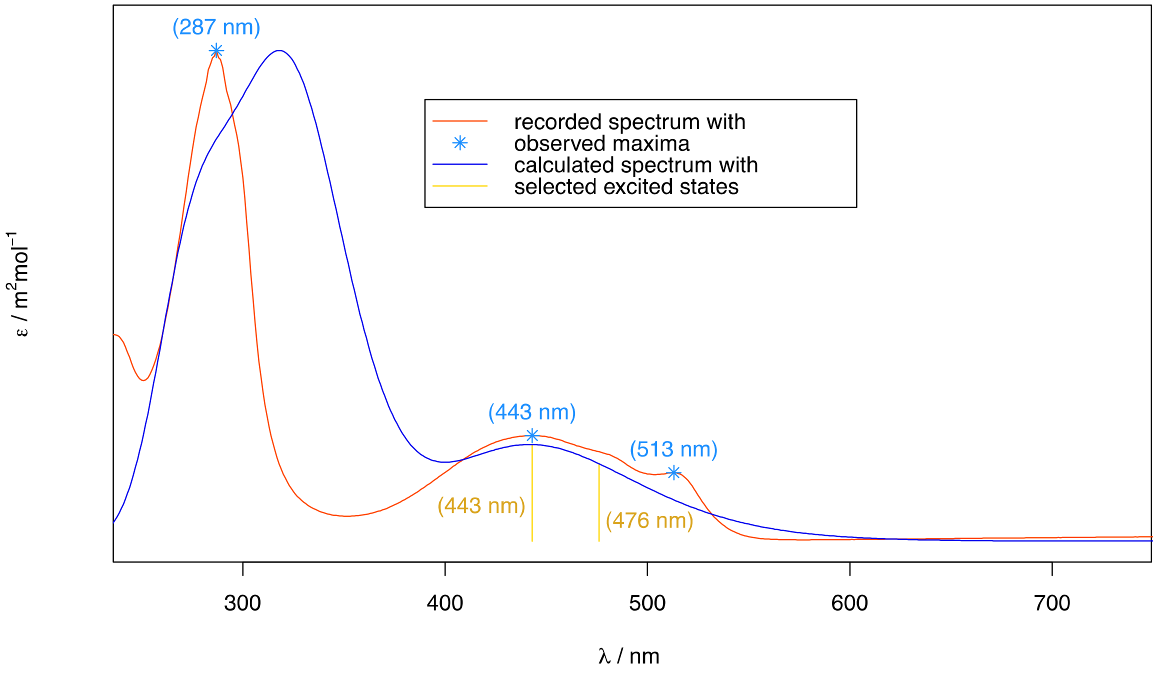

Figure S1. Bottom: Recorded (red) *vs.* calculated (blue) spectrum of **Litrop** in THF. The spectra were normalized at their respective maxima. Top: Involved NTOs (natural transition orbitals) at isovalues of 0.03 in the selected excited states.

**Excited state**	**1**	**2**	**4**
**λ/nm**	**1056.98**	**555.91**	**460.95**
**Oscillator strength**	**0.0027**	**0.0255**	**0.1864**
	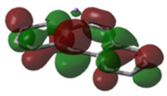	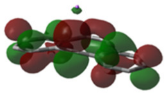	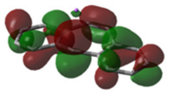
involved NTOs (iso value = 0.03)	56 (HOMO)	56 (HOMO)	56 (HOMO)
	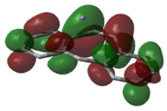	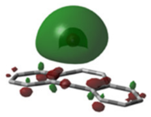	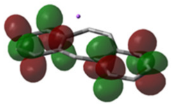
	57 (LUMO)	58 (LUMO+1)	60 (LUMO+3)

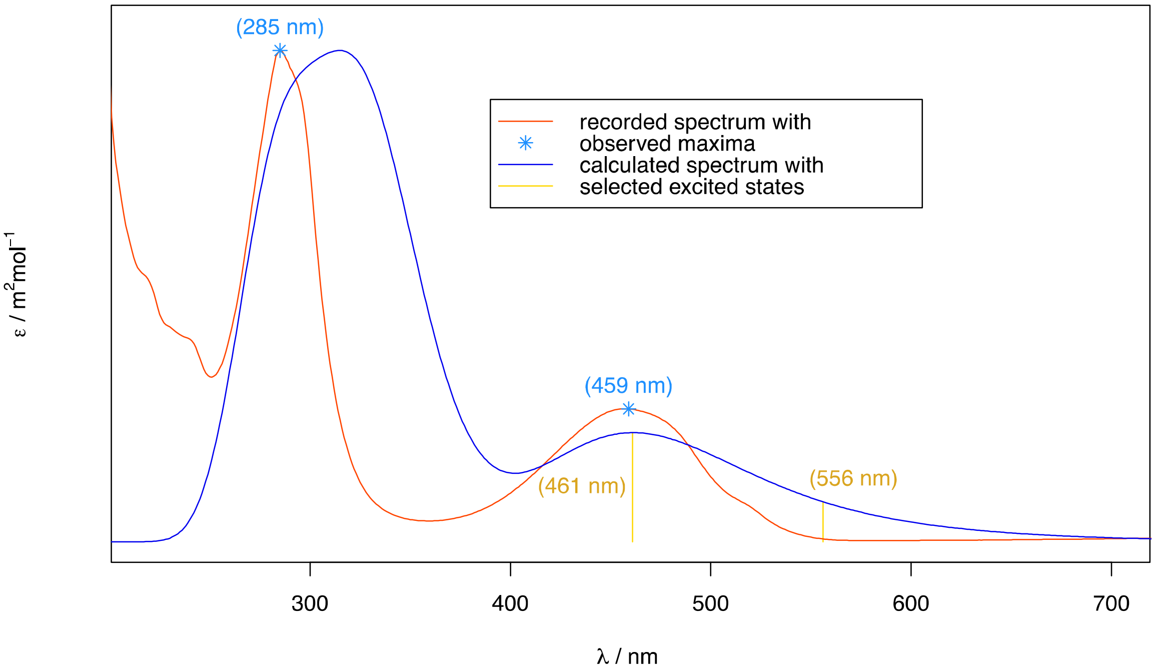

Figure S2. Bottom: Recorded (red) *vs.* calculated (blue) spectrum of **Natrop** in THF. The spectra were normalized at their respective maxima. Top: Involved NTOs (natural transition orbitals) at isovalues of 0.03 in the selected excited states.

**Excited state**	**1**	**4**
**λ/nm**	**1285.89**	**482.89**
**Oscillator strength**	**0.0038**	**0.1957**
involved NTOs (iso value = 0.03)	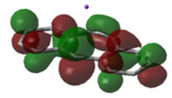<br>60 (HOMO)<br>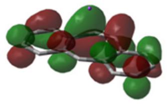<br>61 (LUMO)	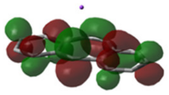<br>60 (HOMO)<br>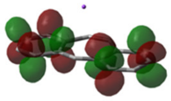<br>64 (LUMO+3)

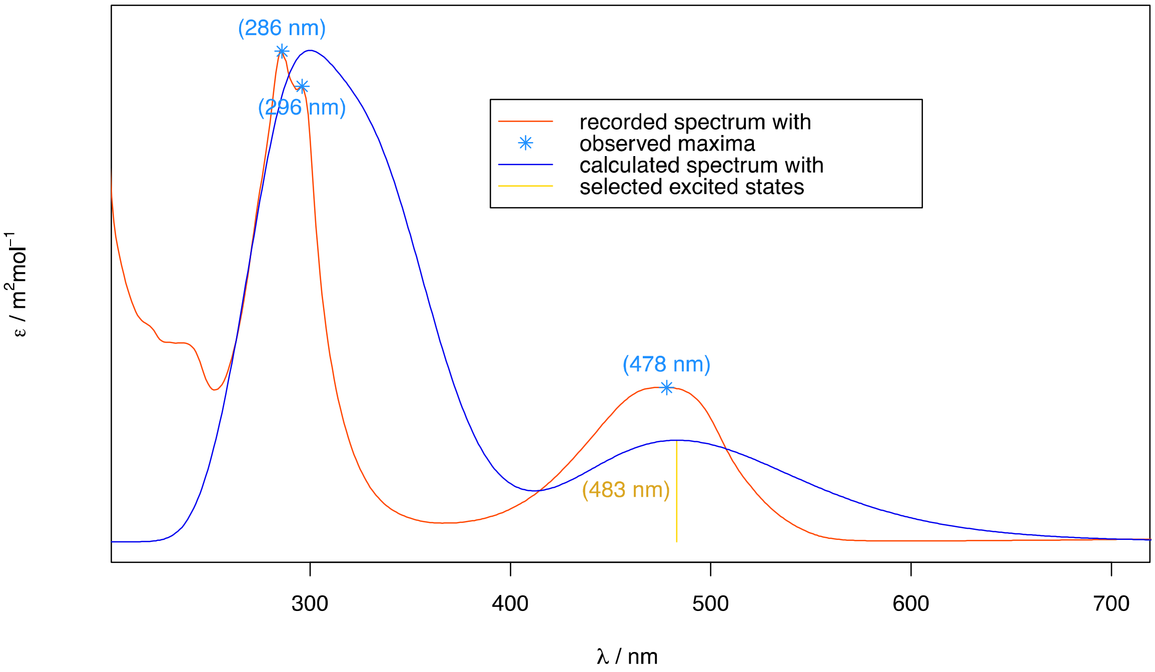

Figure S3. Bottom: Recorded (red) *vs.* calculated (blue) spectrum of **Ktrop** in THF. The spectra were normalized at their respective maxima. Top: Involved NTOs (natural transition orbitals) at isovalues of 0.03 in the selected excited states.

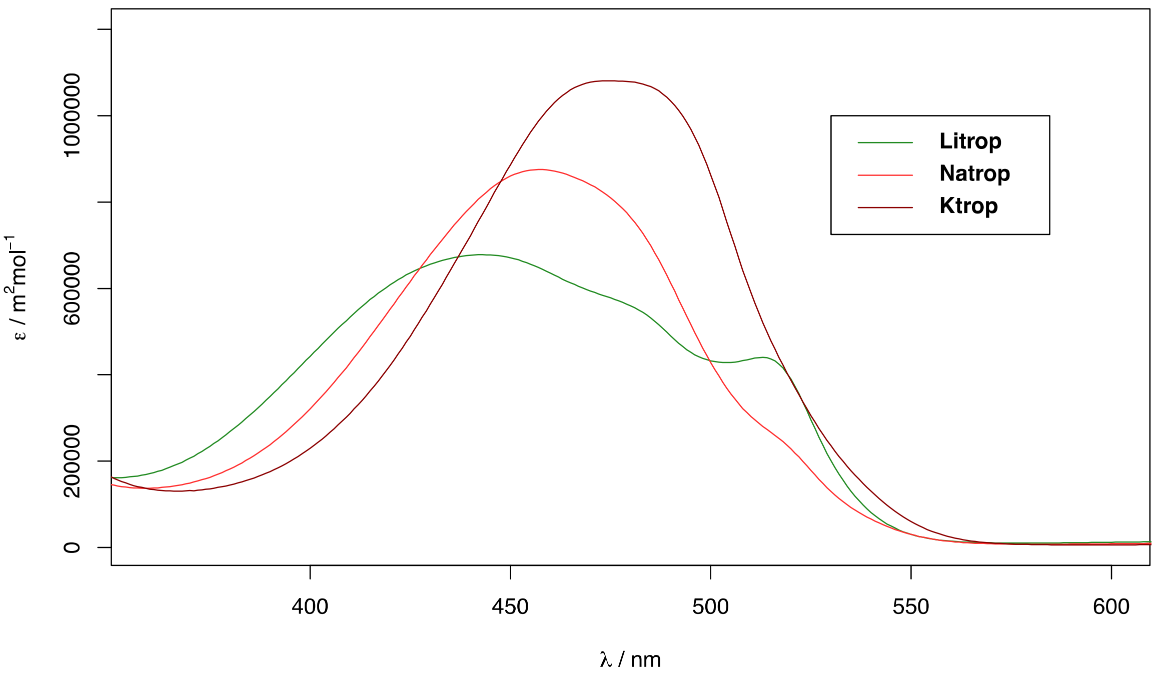

Figure S4. Stacked spectra of **Litrop**, **Natrop** and **Ktrop** in THF.

**Excited state**	**3**	**23**
**λ/nm**	**819.10**	**501.75**
**Oscillator strength**	**0.0116**	**0.1095**
involved NTOs (iso value = 0.03)	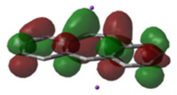 70A (SOMO)	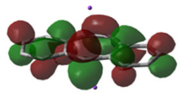 69 (SOMO-1)
	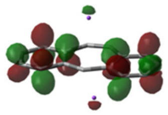 72A (LUMO+1)	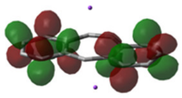 73 (LUMO+2)

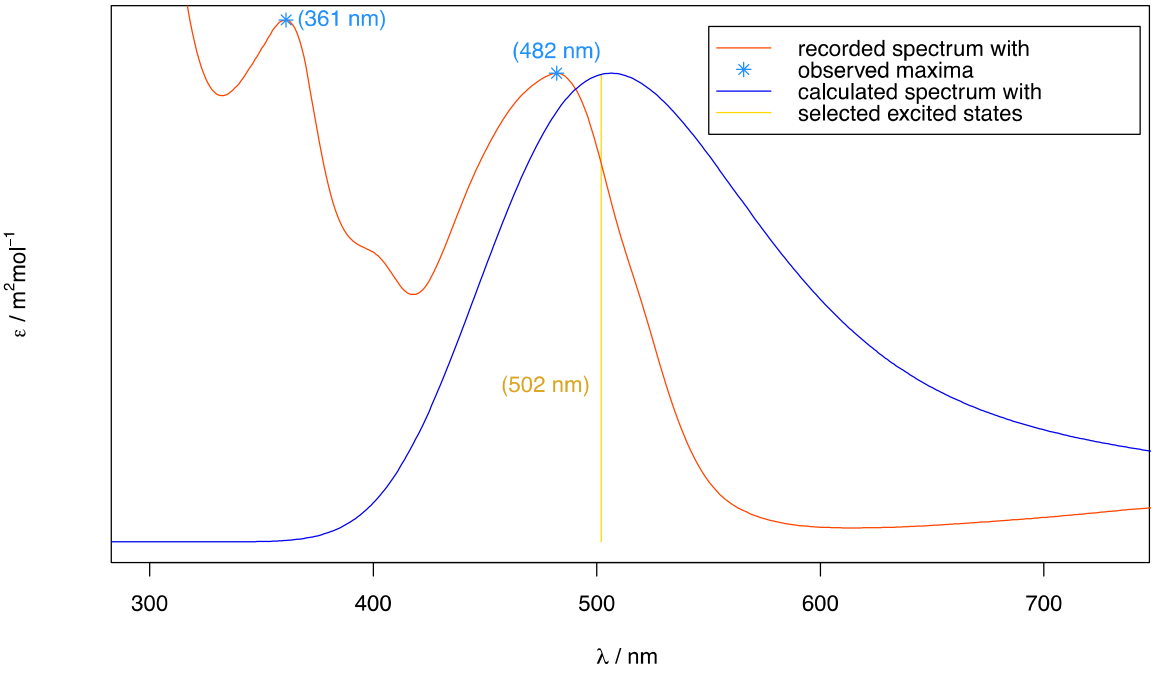

Figure S5. Bottom: Recorded (red) *vs.* calculated (blue) spectrum of **K$_2$trop** in THF. The recorded and the calculated spectra were normalized at 482 nm and at its maximum, respectively. Top: Involved NTOs (natural transition orbitals) at isovalues of 0.03 in the selected excited states.

**Excited state**<br>***λ*/nm**<br>**Oscillator strength**	**1**<br>**638.60**<br>**0.0000**	**2**<br>**608.63**<br>**0.0162**	**22**<br>**336.19**<br>**1.3465**	**26**<br>**319.12**<br>**0.4045**
	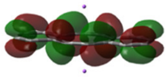	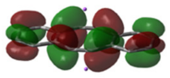	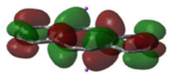	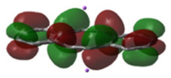
involved NTOs	65 (HOMO)	65 (HOMO)	64 (HOMO-1)	64 (HOMO-1)
	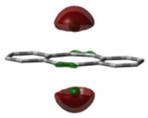	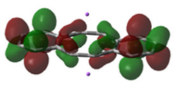	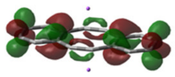	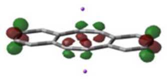
	66 (LUMO)	67 (LUMO+1)	69 (LUMO+3)	72 (LUMO+6)

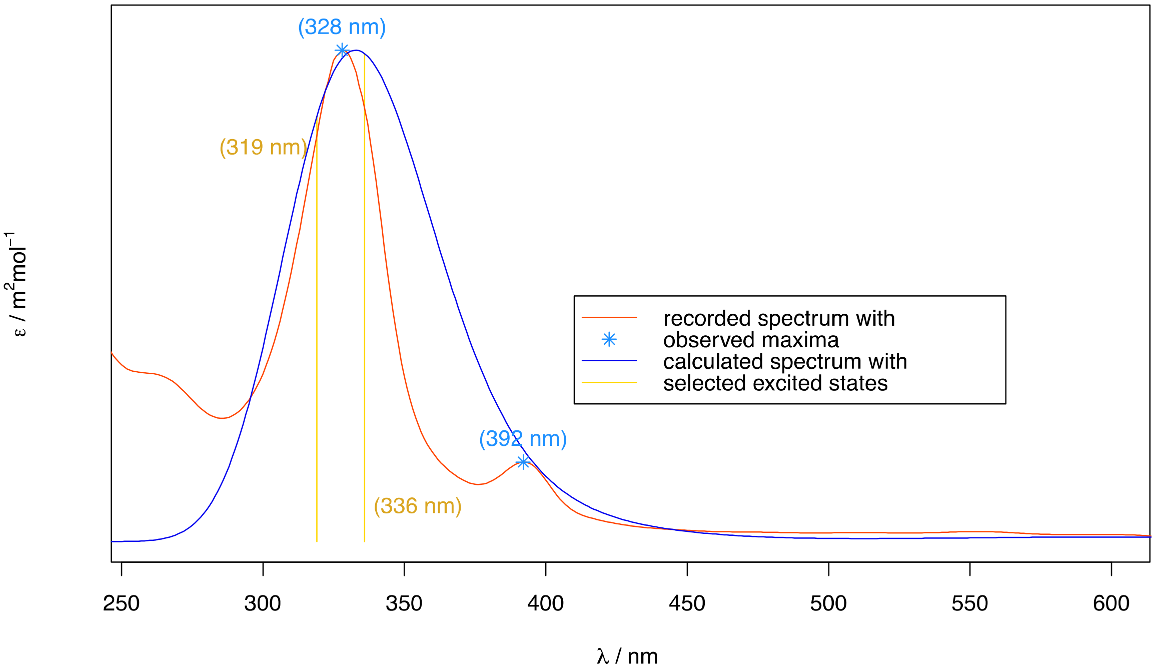

Figure S6. Recorded (red) *vs.* calculated (blue) spectrum of **Na₂dbcot** in THF. The spectra were normalized at their respective maxima. Top: Involved NTOs (natural transition orbitals) at isovalues of 0.03 in the selected excited states.

**Excited state**	**1**	**2**	**25**
**λ/nm**	**610.26**	**357.72**	**334.07**
**Oscillator strength**	**0.0133**	**0.1657**	**1.4731**
	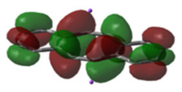	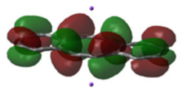	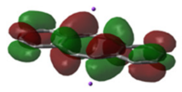
involved NTOs (iso value = 0.03)	73 (HOMO)	73 (HOMO)	73 (HOMO)
	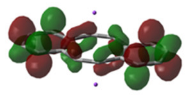	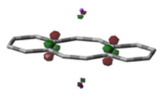	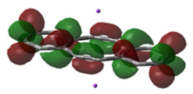
	74 (LUMO)	90 (LUMO+16)	93 (LUMO+19)

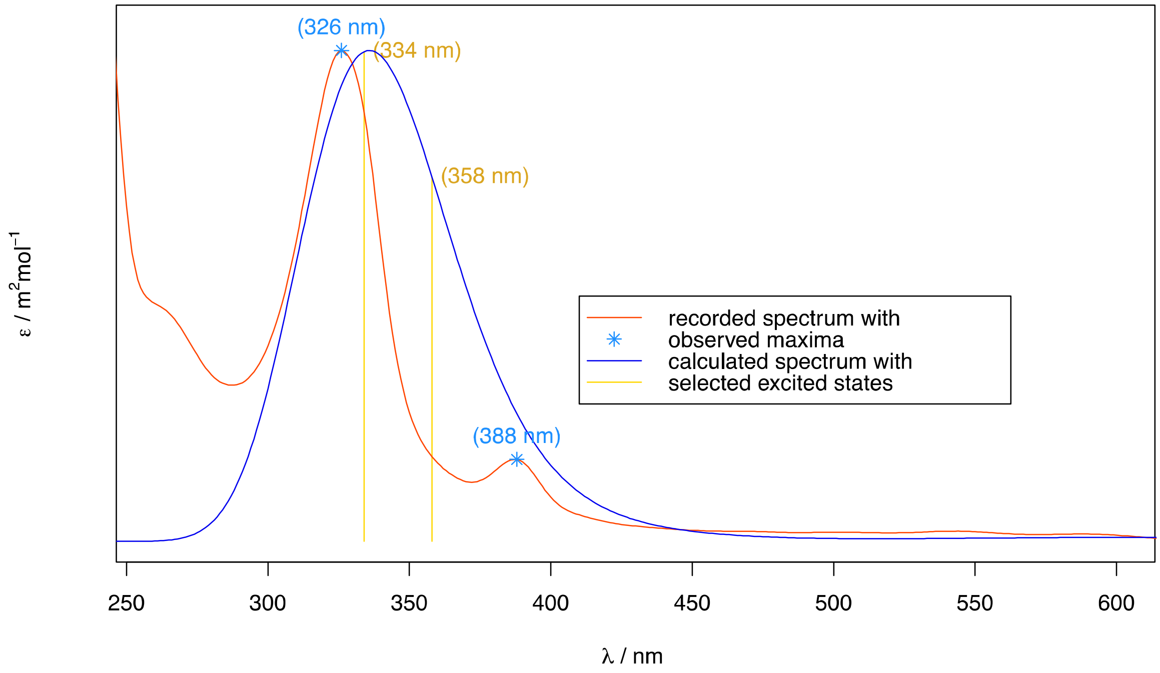

Figure S7. Recorded (red) *vs.* calculated (blue) spectrum of **K_2dbcot** in THF. The recorded and the calculated spectra were normalized at 326 nm and at its maximum, respectively. Top: Involved NTOs (natural transition orbitals) at isovalues of 0.03 in the selected excited states.

2. Electrochemical data for Ktrop and trop$_2$ on GC electrode.

Procedures: All cyclic voltammetry (CV) experiments are performed under strictly anaerobic environments in a conventional three electrode single-compartment cell with a SP 300 Bio-Logic potentiostat (Bio-Logic Science Instruments SAS). A standard Ag/AgNO$_3$ (10 mM) electrode is used as the reference electrode. The reference electrode is calibrated with internal Fc oxidation potential. The average standard potential of Fc/Fc$^+$ redox couple is determined to be 0.287 V and hence is used as correction factor in conversion to Fc/Fc$^+$ reference. Glassy carbon electrodes (GCEs, from BASi) with 3 mm diameter is used as working electrode. Electrodes were polished on wet polishing cloths using a 1 μm diamond suspension and a 0.05 μm alumina slurry. The scan rate was 100 mVs^{-1}. Dry THF was used as solvent and recrystallized TBAPF$_6$ is used for all electrochemical experiments. In general case 1 mM concentration of catalyst is used if not noted otherwise. In all the data 2nd scan of three successive scans is presented.

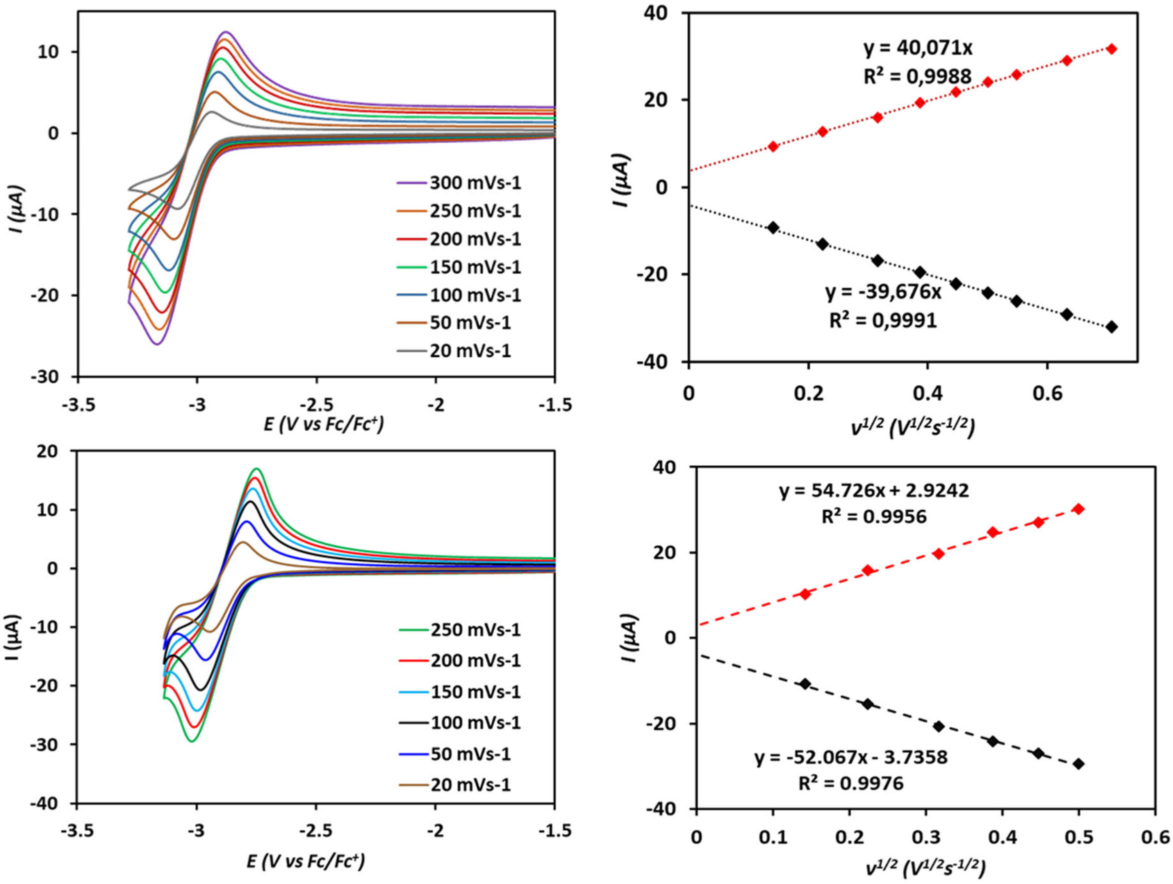

Figure S8. Scan rate dependence of the 1st reduction process for **Ktrop** (top) and **trop$_2$** (bottom) in 0.1 M TBAPF$_6$ solution in THF on glassy carbon electrode. The CV responses are given in left and corresponding Randles-Sevcik plots are given on the right.

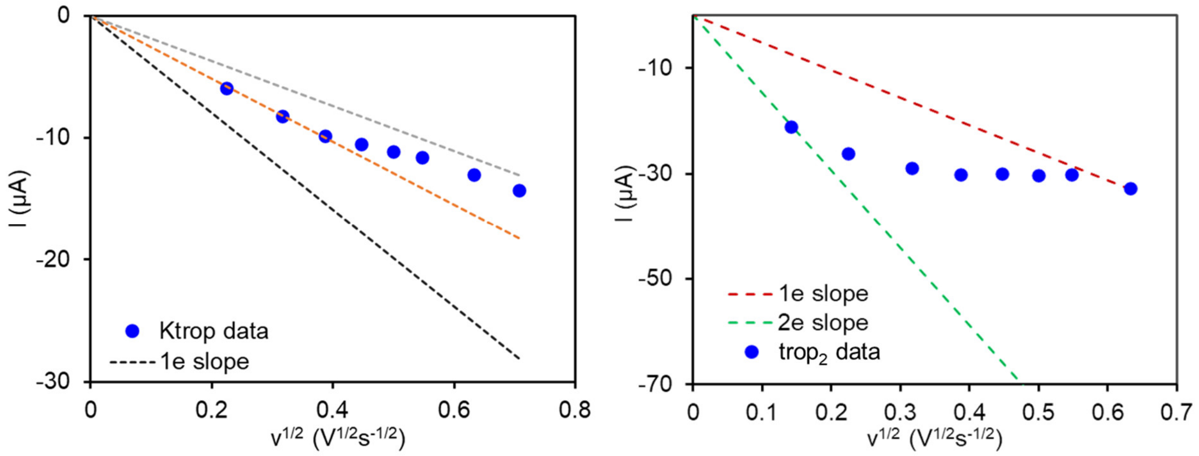

Figure S9. Overlay of the I_p currents (blue dots) *vs.* scan rate$^{1/2}$ plot of **Ktrop$^{2-/3-}$** redox process with theoretical 1e$^-$ and 2e$^-$ plot slope (*left*). The similar plot for **trop$_2$$^{1-/2-}$** is presented on the *right.*

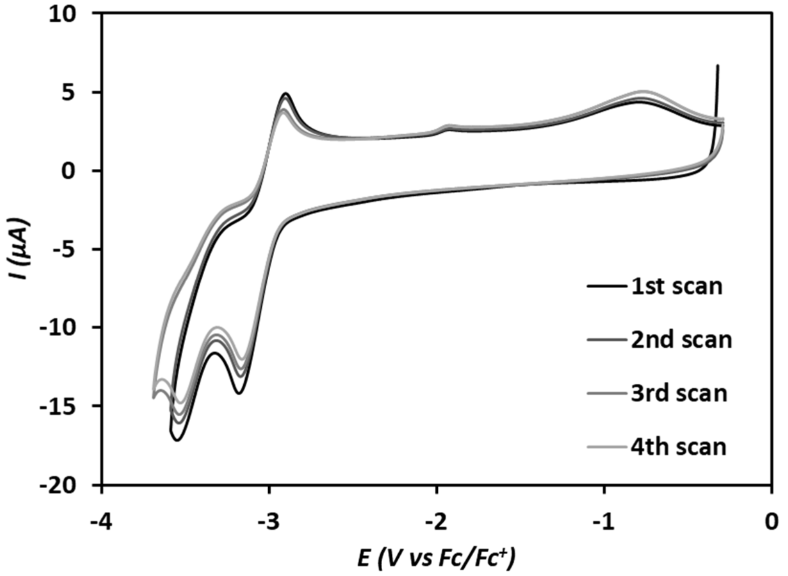

Figure S10. Overlay of the successive CV scans for 1 mM of **Ktrop** solution in THF (0.1 M $TBAPF_6$) at 100 mVs^{-1}.

Determination of the number of electrons associated to the redox events [5]

The number of electrons exchanged during the reduction of **trop$_2$** at $E_{1/2}$ = -2.86 V *vs.* Fc/Fc^+ was determined by the combination of a chronoamperometry experiment and a study of the stationary regime obtained at a carbon microelectrode. Solutions of **trop$_2$** and ferrocene (10 mM in THF with 0.1 M $TBAPF_6$) were prepared. A chronoamperometry study was carried out on both solutions using exactly identical experimental conditions (with glassy carbon as working electrode with 3 mm diameter, platinum wire counter electrode and $Ag/AgNO_3$ reference electrode that is converted to Fc/Fc^+). The plot of the current I versus $t^{-1/2}$ (cf. Figure S11) obey the Cottrell equation [6]:

$$I = kt^{-1/2} \text{ with } k = nFACD^{1/2}(\pi)^{-1/2} \quad (1)$$

where I is the current, n the number of electrons, F the Faraday constant, A is the electroactive area of the electrode, D the diffusion coefficient of the species, C the concentration of the compound and t the time. As the concentration of the ferrocene solution is equal to the one of the trop$_2$, and as the number of electrons involved in the oxidation of ferrocene is 1, the ratio of the director coefficient k of the plot measured for the ferrocene and the trop$_2$ solutions is:

$$k_{trop2}/k_{Fc} = n(D_{trop2}/D_{Fc})^{1/2} \quad (2)$$

where D_{Fc} is the diffusion coefficient for ferrocene, D_{trop2} is the diffusion coefficient for **trop**$_2$ and n is the number of electrons involved in its reduction.

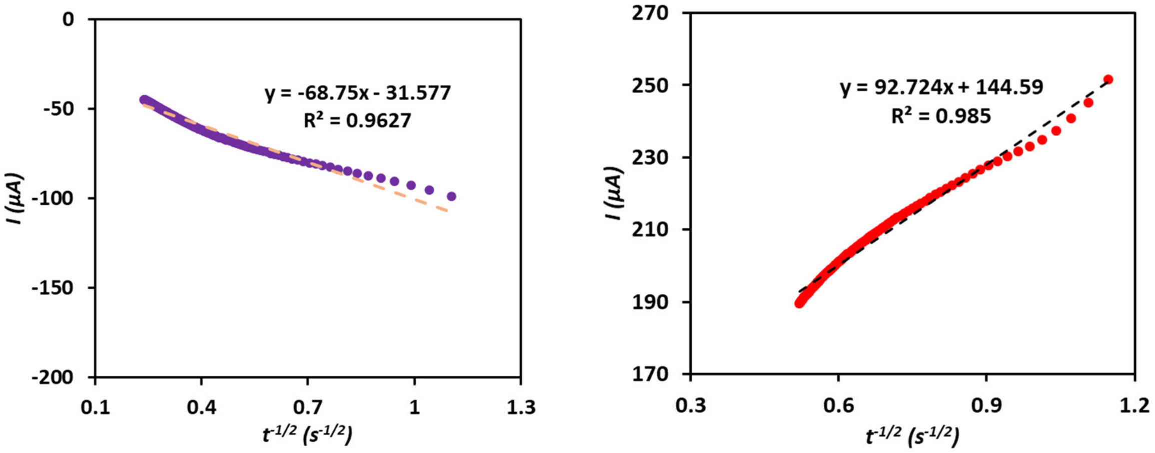

Figure S11. I *vs.* $t^{-1/2}$ plot of the chronoamperometry data obtained from a 10 mM **trop**$_2$ (in purple, left) solution at -3.07 V *vs.* Fc/Fc$^+$ and a 10 mM ferrocene solution (in red, right) at 0.23 V *vs.* Fc/Fc$^+$ in 0.1 M TBAPF$_6$ in THF. In parallel, the intensity of the current at a microelectrode in stationary regime was measured for both solutions (cf. Figure S12). The following relation gives the value of the limiting current:.

$$i^{lim} = 4nFrCD \quad (3)$$

Where i is the current, n the number of electrons, F the Faraday constant, D the diffusion coefficient of the species, r the radius of the carbon microelectrode and C the concentration of the compound in the bulk solution.

Similarly, the ratio of the limit intensities measured for the ferrocene and trop$_2$ solution is:

$$i^{lim}_{trop2}/i^{lim}_{Fc} = nD_{trop2}/D_{Fc} \quad (4)$$

where D_{Fc} is the diffusion coefficient for ferrocene, D_{trop2} is the diffusion coefficient for trop$_2$ and n is

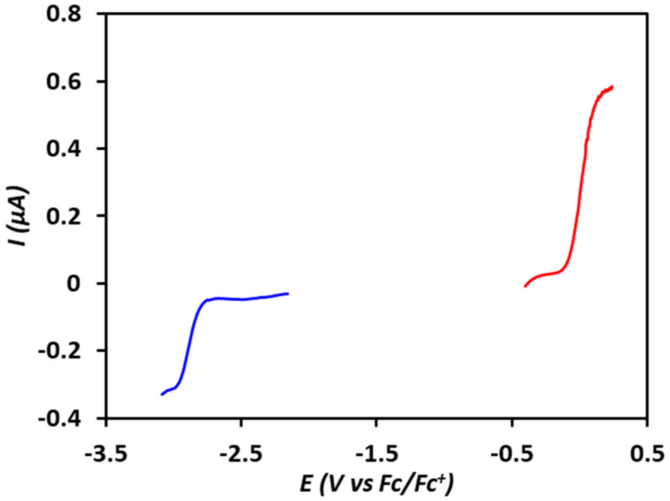

the number of electrons involved in its reduction at $E_{1/2}$ = -2.27 V *vs.* Fc/Fc$^+$.

Figure S12. Overlay of linear sweep voltammograms of a 10 mM **trop**$_2$ solution (blue) and a 10 mM ferrocene (red) in a 0.1 M TBAPF$_6$ solution in THF obtained in stationary regime at a carbon microelectrode (5 mVs^{-1} scan rate).

The number of electrons exchanged during the reduction of **trop2** is then given by:

$$n = (k_{Cpx}/k_{Fc})^2/(i^{lim}_{Cpx}/i^{lim}_{Fc}). \quad (5)$$

Table S2. Key parameters for the determination of the number of electrons.

K_{trop2} (μA.s$^{1/2}$)	k_{Fc} (μA.s$^{1/2}$)	I^{lim}_{trop2} (μA)	I^{lim}_{Fc} (μA)	n
68.75	92.724	0.267	0.525	**1.08**

From our electrochemical data (cf. **Error! Reference source not found.**), we find **n = 1.08**. Accordingly, the process observed by electrochemistry for the reduction of trop2 at $E_{1/2}$ = -2.86 V *vs.* Fc/Fc$^+$ involves a transfer of **1 electron**.

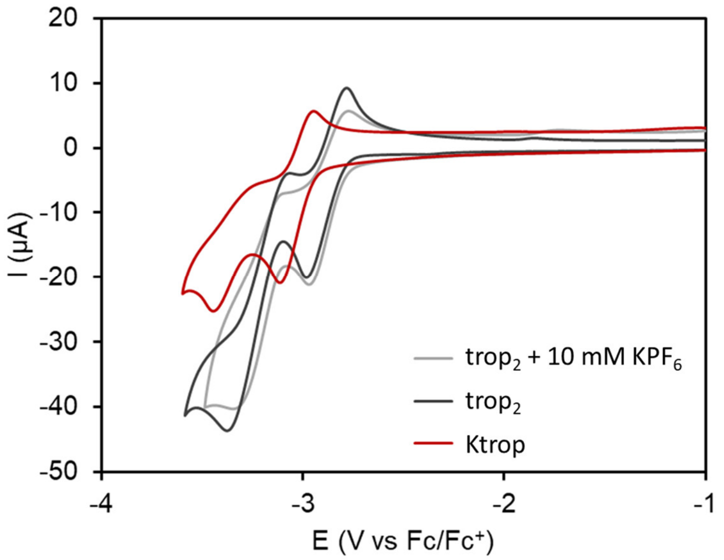

Figure S13. Overlay of the CVs recorded for **Ktrop** (red), **trop2** (black) and **trop2** in presence of 10 mM KPF_6 (grey).

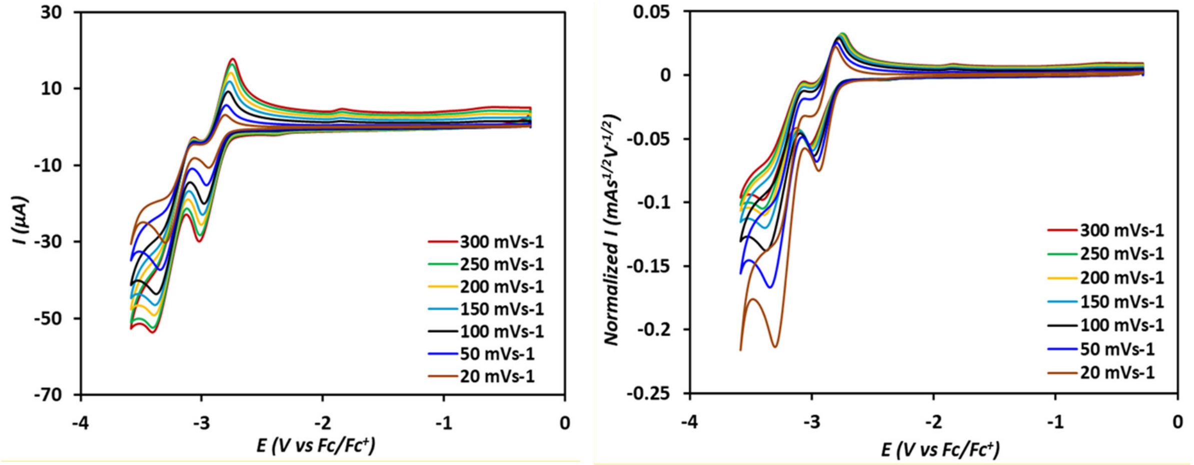

Figure S14. Left: Overlay of the cyclic voltammograms at various scan rates with 1 mM **trop2** in 0.1 M $TBAPF_6$ solution in THF on glassy carbon electrode. Right: Corresponding normalized current plot where the normalization is performed with respect to scan rates.

3. Reactivity studies

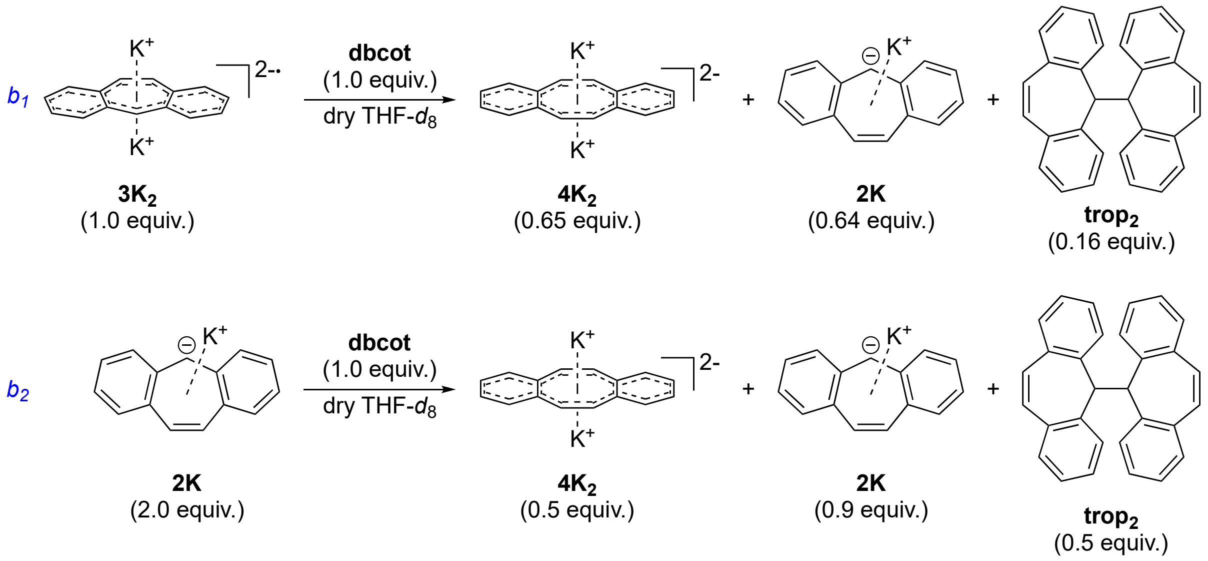

Scheme S1: Further interconversion reactions between the trop and the dbcot scaffold.

References

1. Frisch, M. J.; Trucks, G. W.; Schlegel, H. B.; Scuseria, G. E.; Robb, M. A.; Cheeseman, J. R.; Scalmani, G.; Barone, V.; Petersson, G. A.; Nakatsuji, H.; Li, X.; et al. *Gaussian 09 Rev. D.01*, Wallingford, CT, 2013.
2. Perdew, J. P.; Burke, K.; Ernzerhof, M., Generalized Gradient Approximation Made Simple. *Phys. Rev. Lett.* **1997,** *78* , 1396-1396.
3. Perdew, J. P.; Burke, K.; Ernzerhof, M., Generalized Gradient Approximation Made Simple. *Phys. Rev. Lett.* **1996,** *77*, 3865-3868.
4. Krishnan, R.; Binkley, J. S.; Seeger, R.; Pople, J. A., Self-consistent molecular orbital methods. XX. A basis set for correlated wave functions. *J. Chem. Phys.* **1980,** 72, 650-654.
5. Camp, C.; Guidal, V.; Biswas, B.; Pécaut, J.; Dubois, L.; Mazzanti, M., Multielectron redox chemistry of lanthanide Schiff-base complexes. *Chem. Sci.* **2012,** 3, 2433-2448.
6. Bard, A. J.; Faulkner, L. R., *Electrochemical Methods: Fundamentals and Applications*. John Wiley: New York, 2001.

© 2020 by the authors. Licensee MDPI, Basel, Switzerland. This article is an open access article distributed under the terms and conditions of the Creative Commons Attribution (CC BY) license (http://creativecommons.org/licenses/by/4.0/).
